# Direct nucleophilic trifluoromethylation of carbonyl compounds by potent greenhouse gas, fluoroform: Improving the reactivity of anionoid trifluoromethyl species in glymes

**DOI:** 10.1038/s41598-018-29748-1

**Published:** 2018-07-31

**Authors:** Takuya Saito, Jiandong Wang, Etsuko Tokunaga, Seiji Tsuzuki, Norio Shibata

**Affiliations:** 1 Department of Nanopharmaceutical Sciences, Nagoya Institute of Technology, Gokiso, Showa-ku, Nagoya, 466-5888 Japan; 2 Research Center for Computational Design of Advanced Functional Materials, AIST, Tsukuba, Ibaraki, 305-8568 Japan; 3 Institute of Advanced Fluorine-Containing Materials, Zhejiang Normal University, 688 Yingbin Avenue, 321004 Jinhua, China

## Abstract

A simple protocol to overcome the problematic trifluoromethylation of carbonyl compounds by the potent greenhouse gas “HFC-23, fluoroform” with a potassium base is described. Simply the use of glymes as a solvent or an additive dramatically improves the yields of this transformation. Experimental results and DFT calculations suggest that the beneficial effect deals with glyme coordination to the K^+^ to produce [K(polyether)_n_]^+^ whose diminished Lewis acidity renders the reactive anionoid CF_3_ counterion species more ‘naked’, thereby slowing down its undesirable decomposition to CF_2_ and F^−^ and simultaneously increasing its reactivity towards the organic substrate.

## Introduction

**Figure 1:**
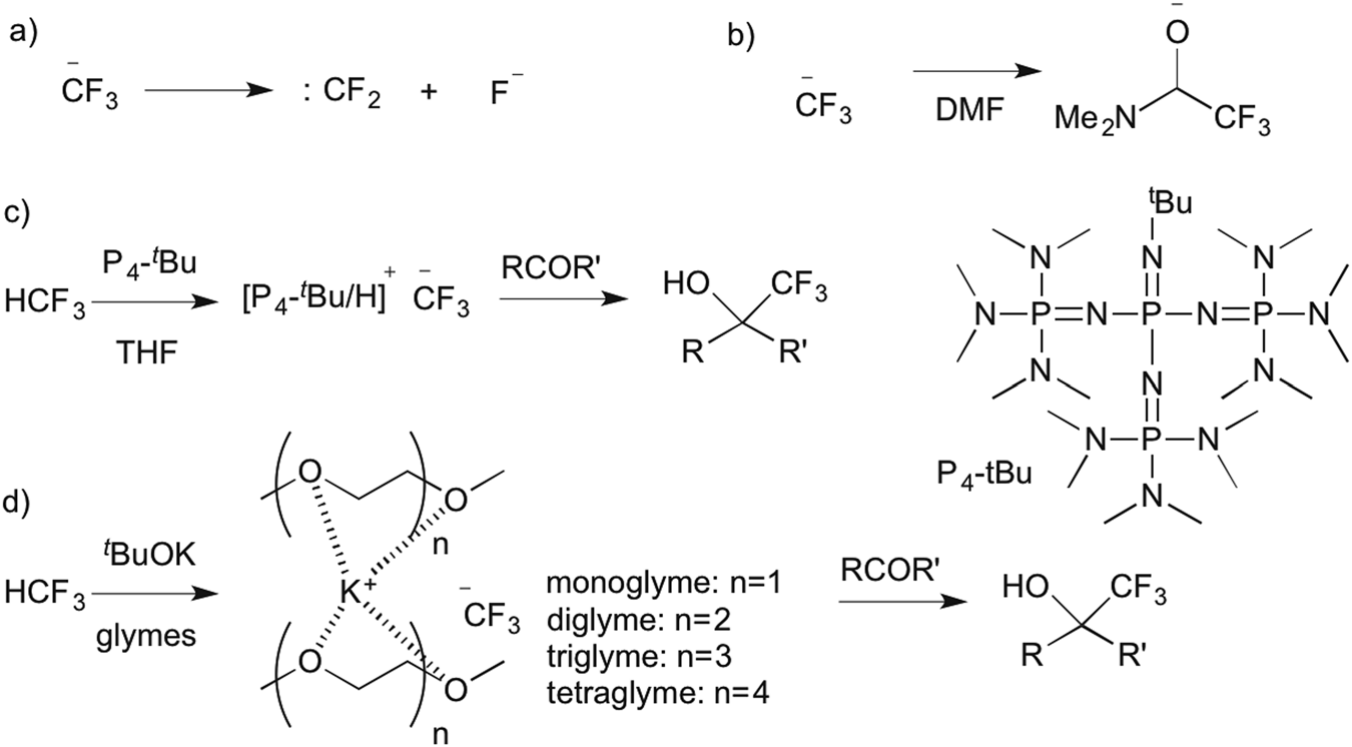
(**a**) Decomposition of CF_3_ anion to difluorocarbene (:CF_2_) and fluoride (F^−^). (**b**) Stabilization of CF_3_ anion by DMF. (**c**) Direct trifluoromethylation of carbonyl compounds by using sterically demanding P4-^*t*^Bu base. (**d**) Encapsulation of K^+^ with glymes for trifluoromethylation (this work).

There has been remarkable progress recently in the synthetic incorporation of a trifluoromethyl (CF_3_) moiety into potential bioactive molecules, prompting the discovery of new pharmaceuticals and agrochemicals^1–5^. Fluoroform (HFC-23, HCF_3_, trifluoromethane) is a potent greenhouse gas that is formed as a by-product in huge amounts during the synthesis of poly-tetrafluoroethylene (PTFE) and polyvinylidene difluoride (PVDF) from chlorodifluoromethane (ClCHF_2_). Fluoroform has a 11,700-fold higher GWP than carbon dioxide with an atmospheric lifetime of 264 years and is used to a very limited extent as a refrigerant or as a raw material^6–10^. At present, fluoroform abatement techniques involve thermal oxidation, catalytic hydrolysis and plasma destruction, so there are operation and economical limits to transform fluoroform to useful refrigerants or fire extinguishers^11–17^. HFC-23 is an easily handled, stable and non-toxic trifluoromethyl (CF_3_) source^18–24^. Thus the synthetic use of HCF_3_ serving as feedstock for various trifluoromethylations is highly desirable. However, chemoselective and efficient activation of HCF_3_ for nucleophilic trifluoromethylation processes, is a long-standing, challenging and intriguing issue in organic chemistry. One of the primary problems in the extensive usage of HCF_3_ for trifluoromethylations is the facile decomposition of the CF_3_^−^ anion to difluorocarbene (:CF_2_) and fluoride (F^−^)^18^. This decomposition is probably induced by the strong repulsion between the lone electron pairs on the carbon and fluorine atoms of CF_3_^−^ (Fig. 1a). In the presence of alkali (M^+^) and other metal cations, the decomposition to difluorocarbene is particularly favored due to the formation of highly stable fluoride salts, such as MF.

Several strategies have emerged to use HCF_3_ for trifluoromethylation via deprotonation with strong organic or inorganic bases^18–24^. In 1991, Shono and co-workers for the first time reported the trifluoromethylation of carbonyl compounds with fluoroform by electrogenerated bases as well as common bases such as NaH and ^*t*^BuOK in DMF^19^. Subsequently, Barhdadi, Troupel and Perichon reported the trifluoromethylation of aldehydes with fluoroform by a strong base generated via cathodic reduction of iodobenzene^20^. Then Nomant and Roques demonstrated use of MeSOCH_2_K^21^ and KHMDS^23^ in DMF for trifluoromethylation of carbonyl compounds. It should be pointed out that, in all of these original developments, N,N-dimethylformamide (DMF, Me_2_NCHO) was used as the solvent. The crucial role of DMF was to stabilize the CF_3_^−^ generated on deprotonation of HCF_3_ in the form of the hemiaminaloate [Me_2_NCH(O)CF_3_]^−^, which served as a CF_3_^−^ “reservoir” in the reaction (Fig. 1b). In 2011, Grushin and co-workers reported the direct cupration of fluoroform with the dialkoxycuprate produced from CuCl and ^*t*^BuOK in a 1:2 ratio to prepare CuCF_3_^25–27^, which since then has been successfully applied to a wide variety of trifluoromethylations^28–35^. The cupration of fluoroform is governed by a concerted ambiphilic metal-ligand activation (AMLA) mechanism rather than simple deprotonation to give CF_3_^−^ and/or difluorocarbene intermediates^27^. The important dual effect of the alkali-metal counterion, which would slowly decompose CuCF_3_ via α-fluoride elimination but also provides electrophilic assistance for the CF_3_H cupration, was demonstrated by adding stoichiometric amounts of 18-crown-6 or [2.2.2]crypt and (crypt-222) before and after the cupration, in order to diminish the electrophilicity of alkali-metal cation^25,27^. While the CuCF_3_ is stable, its direct synthesis from HCF_3_ requires an amide solvent, such as DMF, DMA, and NMP.

The first DMF-free trifluoromethylation with HCF_3_ was reported by Langlois and co-workers in 2000^24^. Although a catalytic amount of DMF was still needed for trifluoromethylation of carbonyl compounds with HCF_3_/N(TMS)_3_/[Bu_4_N]^+^ [Ph_3_SiF_2_]^−^ or Me_4_NF/DMF, the trifluoromethylation of dioctyl disulfide was successfully carried out in pure THF (66% yield). In 2012, Prakash *et al*. also reported nucleophilic trifluoromethylations of Si, B, S, and C centers by HCF_3_ using potassium hexamethyldisilazide (KHMDS) in the absence of DMF^36^. The formation of a KCF_3_ intermediate followed by CF_3_ transfer to the organic substrate was proposed in a DFT study^37^. Simultaneously, we reported that a sterically demanding Schwesinger base, phosphazene P4-^*t*^Bu, is effective for pushing inert HCF_3_ to nucleophilic trifluoromethylation of carbonyl compounds, disulfides, and arylsulfonyl fluorides in the absence of DMF and any metals^38,39^. Being metal-free, our HCF_3_/P4-^*t*^Bu system efficiently suppresses the decomposition of CF_3_^−^ to difluorocarbene and fluoride, as explained above (Fig. 1c). Very recently, Szymczak and co-workers reported a new type of Lewis acid-CF_3_ adducts formed from an alkali metal hydride, HCF_3_ and boron-based Lewis acids^40,41^. Although these are important developments, simple, cost-efficient, and environmentally benign methods are needed to perform trifluoromethylation reactions with HCF_3_ on a large scale. We now report a simple protocol for one-step trifluoromethylation of carbonyl compounds with HCF_3_ in the presence of ^*t*^BuOK or KHMDS. While being fundamentally similar to the previously reported methods based on deprotonation of HCF_3_^24,36^, our new protocol features a dramatic improvement from performing the reaction in the presence of a suitable amount of polyethers such as glymes (Fig. 1d). A wide variety of ketones, chalcones and aldehydes are nicely converted to the trifluoromethylated carbinols by HCF_3_ under the optimized glyme conditions. Cyclic polyethers such as 18-crown-6 and crypt-222 are even more effective. The encapsulation of the K^+^ by acyclic or cyclic polyethers is the key for this transformation, which makes the reactive anionoid CF_3_ species more “naked”.

## Results

**Figure 2:**
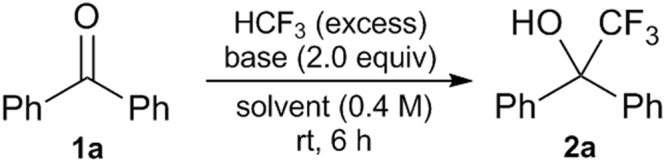
Optimization of reaction conditions of **1a** to **2a** by HCF_3_.

Towards an economical and practical method, we intended to use glymes for tuning the Lewis acidity, hardness and steric bulk of the potassium-based counter-cation to CF_3_^−^^ 42^. Glymes, saturated non-cyclic polyethers, are usually less volatile, miscible with water, and less toxic than many other organic solvents^43^. We initiated our investigation with the reaction of benzophenone (**1a**) and HCF_3_ (excess) with ^*t*^BuOK (2.0 equiv) in THF or 1,2-dimethoxyethane (DME or monoglyme, 0.4 M) at room temperature (rt) for 6 h (runs 1 and 2, Table 1 and Fig. 2). While a desired 2,2,2-trifluoro-1,1-diphenylethan-1-ol (**2a**) was obtained in 52% yield in THF (run 1), a much higher yield of 88% was observed in monoglyme (run 2). This yield (88%) in monoglyme is noticeably higher than in the reported DMF-free reaction employing much more costly KHMDS (71%)^36^ and comparable with our ^*t*^Bu-P4 method (92%)^38^. Encouraged by the initial result, we explored the possibility of using diglyme, triglyme and tetraglyme in this reaction. The yields of **2a** appeared to increase with the size of the glyme (runs 3–5). In triglyme and tetraglyme, the desired product was produced quantitatively (>99%, runs 4 and 5). By using 1.0 equiv of ^*t*^BuOK in triglyme or tetraglyme, the yields were lower, 64% and 74%, respectively (runs 6 and 7). HMDS bases were also examined in triglyme, and only potassium base was effective (runs 8–10). In order to gain more insight into the importance of coordination of triglyme to the K^+^, control experiments were conducted (runs 11–16; see Table S1 for more details). In the absence of triglyme, a 32% yield of **2a** was obtained in toluene with ^*t*^BuOK. Interestingly, a steady increase in the yield (from 54% to >99%) was observed as the amount of triglyme in the system was increased from 1.0 to 4.0 equiv. These results suggested 2:1 coordination of triglyme to K^+^, furnishing the complex cation [K(triglyme)_2_]^+^. While the use of triglyme (4.0 equiv) in toluene was clearly a good choice for the conditions (run 15), for simplicity we selected monoglyme and triglyme as solvents rather than additives (runs 2 and 4). The 2:1 coordination was also confirmed for tetraglyme/K^+^, [K(tetraglyme)_2_]^+^, in a series of similar experiments. The comparison of the amount of HCF_3_ was finally examined (runs 4, 17–20). In principle, one equiv of HCF_3_ was enough for nearly quantitative transformation (runs 4 vs 17), and the slightly lower yield (99% vs 90%) was probably due to the technical issues. Thus, we concluded that one equiv of HCF_3_ is suitable for this transformation. More details of the optimization of the reaction conditions are shown in Table S1.

**Table 1:** Optimization of reaction conditions of **1a** to **2a** by HCF_3_.

run	solvent	base	additive (equiv)	yield (%)^*^	run	solvent	base	additive (equiv)	yield (%)^*^
1	THF	^*t*^BuOK		52	11	toluene	^*t*^BuOK		32
2	monoglyme	^*t*^BuOK		88	12	toluene	^*t*^BuOK	triglyme (1.0)	54
3	diglyme	^*t*^BuOK		94	13	toluene	^*t*^BuOK	triglyme (2.0)	74
4	triglyme	^*t*^BuOK		>99	14	toluene	^*t*^BuOK	triglyme (3.0)	86
5	tetraglyme	^*t*^BuOK		>99	15	toluene	^*t*^BuOK	triglyme (4.0)	>99
6	triglyme	^*t*^BuOK (1.0 equiv)		64	16	toluene	^*t*^BuOK	triglyme (5.0)	>99
7	tetraglyme	^*t*^BuOK (1.0 equiv)		74	17^†^	triglyme	^*t*^BuOK		90
8	triglyme	KHMDS		90	18^‡^	triglyme	^*t*^BuOK		92
9	triglyme	LiHMDS		0	19^§^	triglyme	^*t*^BuOK		>99
10	triglyme	NaHMDS		0	20^||^	triglyme	^*t*^BuOK		>99

^*19^F NMR yield with PhCF_3_ as internal standard.

^†^HCF_3_ (1.0 equiv) was used. ^‡^HCF_3_ (2.0 equiv) was used.

^§^HCF_3_ (3.0 equiv) was used. ^||^HCF_3_ (6.0 equiv) was used.

**Figure 3:**
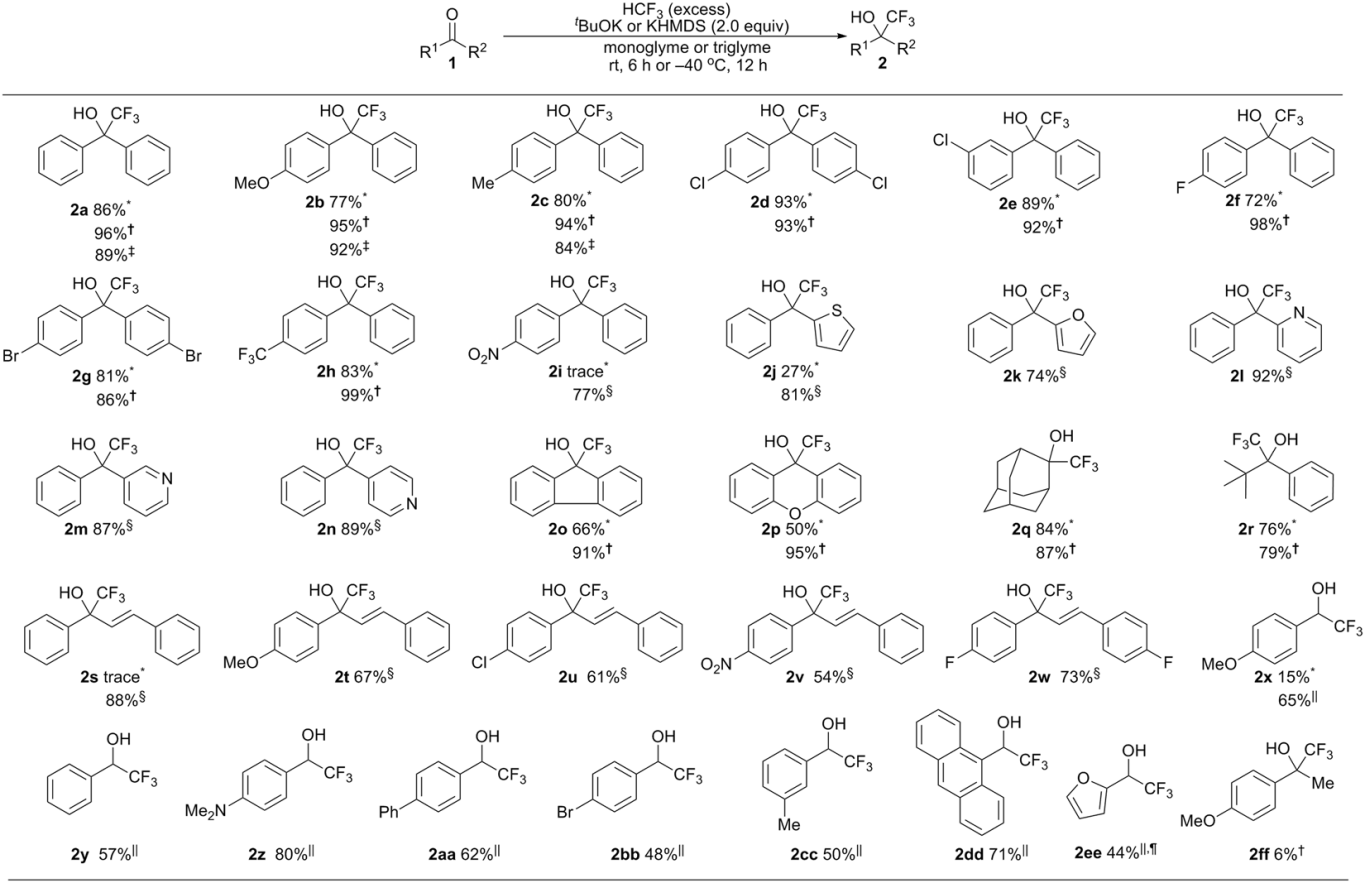
Substrate scope of trifluoromethylation of ketones, chalcones and aldehydes by HCF_3_ in the presence of ^*t*^BuOK or KHMDS in monoglyme or triglyme. *The reaction of **1** (0.2 mmol) with HCF_3_ (excess) was carried out in the presence of ^*t*^BuOK (2.0 equiv) in monoglyme (0.4 M) at rt; ^†^The reaction of **1** (0.2 mmol) with HCF_3_ (excess) was carried out in the presence of ^*t*^BuOK (2.0 equiv) in triglyme (0.4 M) at rt; ^‡^The reaction of **1** (0.2 mmol) with HCF_3_ (1.0 equiv) was carried out in the presence of ^*t*^BuOK (2.0 equiv) in triglyme (0.4 M) at rt; ^§^The reaction of **1** (0.2 mmol) with HCF_3_ (excess) was carried out in the presence of KHMDS (2.0 equiv) in triglyme (0.2 M) at −40 °C; ^||^The reaction of 1 (0.2 mmol) with HCF_3_ (excess) was carried out in the presence of ^*t*^BuOK (2.0 equiv) in triglyme (0.2 M) at −40 °C; ^¶19^F NMR yield.

The substrate generality of this process in monoglyme or triglyme was next investigated using a variety of ketones, chalcones and aldehydes (Fig. 3). While one equiv of HCF_3_ is enough for the almost quantitative transformation (run 17, Table 1), we carried out the reaction mainly by using HCF_3_ in excess for simplicity. A series of diaryl ketones **1a**–**h** with a variety of substituents on the aromatic rings, such as methyl, methoxyl, chloro, bromo and trifluoromethyl groups, were smoothly converted to corresponding trifluoromethyl carbinols **2a**–**h** in good to excellent yield (72–93%) in monoglyme (0.4 M) and in nearly quantitative yield (up to 99%) in triglyme (0.4 M) at rt. For cyclic diaryl ketones **1o** and **1p**, a noticeable increase in the yield was observed in triglyme. Slightly better yields were also detected for bulky aliphatic-substituted ketones **1q** and **1r**. As for the nitro-substituted ketone **1i** and heteroaryl substrates **1j**–**n**, the transformation was less efficient, possibly due to coordination with potassium to the NO_2_ group and to the heteroatoms of the substrate. After further brief screenings of the reaction conditions (see Tables S2 and S3), the desired trifluoromethylated products **2i**–**n** were obtained in high yields (77–91%) under modified reaction conditions employing KHMDS (2.0 equiv) as the base at −40 °C for 12 hours. Subsequently, several chalcones **1s**–**w** with electron-donating and electron-withdrawing substituents on the aryl ring were also converted to the corresponding products **2s**–**w** in 54–88% yields under such conditions. Aromatic aldehydes were found to be compatible with the reaction conditions using triglyme and ^*t*^BuOK to produce the corresponding products **2x**–**2ee** in 44–80% yields. The diminished yield in some cases might be due to side processes, such as the Cannizaro reaction. Unfortunately, only 6% of product **2ff** was obtained in the reaction of **1ff** bearing an enolizable α-proton. To demonstrate the scalability of the method, trifluoromethyl carbinol **2a** was synthesized from benzophenone **1a** (1.822 g, 10.0 mmol) in 93% isolated yield under the standard triglyme reaction conditions.

**Figure 4:**
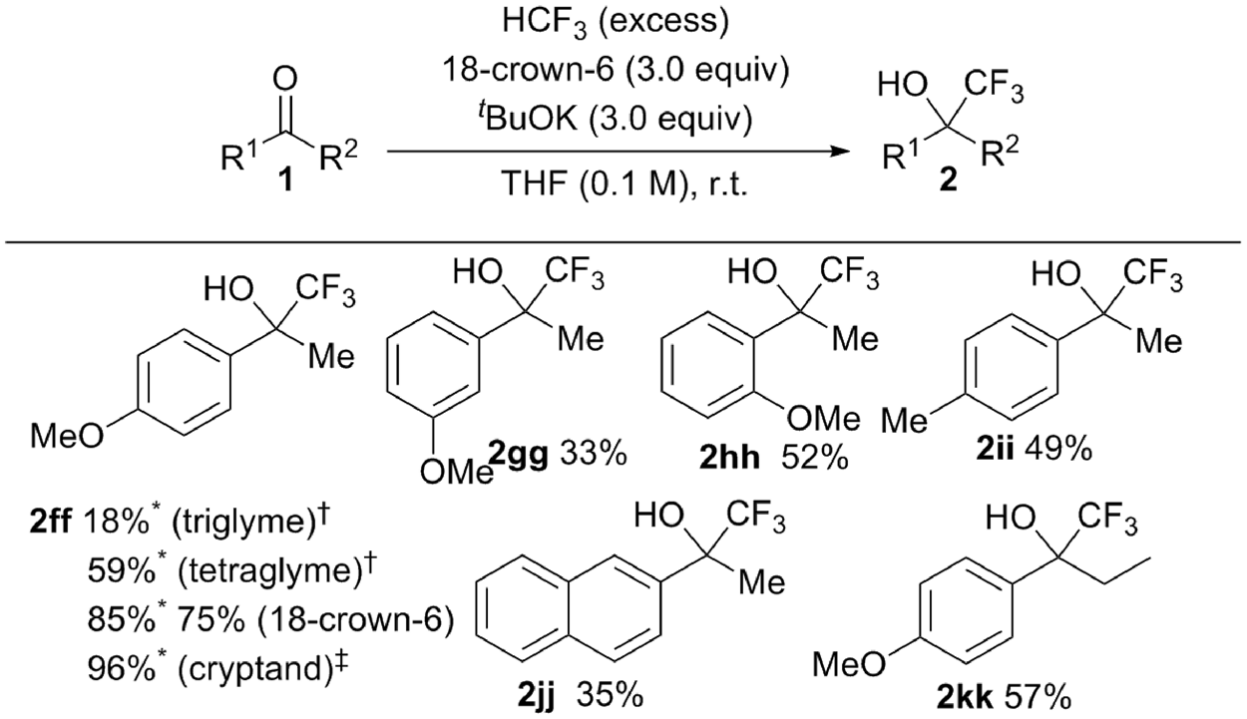
Trifluoromethylation of enolizable ketones **1ff**–**kk** by HCF_3_ with 18-crown-6. ^*19^F NMR yield; ^†^The reaction of **1ff** with HCF_3_ (excess) was carried out in the presence of ^*t*^BuOK (3.0 equiv) in glymes (0.1 M, triglyme or tetraglyme) at rt; ^‡^Crypt-222 was used.

The trifluoromethylation of enolizable ketones such as **1ff** could be improved by reducing the Lewis acidity of the counter cation K^+^ with more powerful ligands. Tetraglyme and cyclic ethers were further considered. After additional optimization of the reaction conditions (Table S4), **1ff** was converted to the desired trifluoromethylated product **2ff** in moderate to good yields, up to 96% depending on ligand used (triglyme, tetraglyme, 18-crown-6, crypt-222; Fig. 4). The yield of **2ff** clearly increased with stronger ligation of the K^+^ prompting a weakening in its Lewis acidity in the order: [K(triglyme)_2_]^+^ > [K(tetraglyme)_2_]^+^ > [K(18-crown-6)]^+^ > [K(crypt-222)]^+^. Substrate generality of enolizable ketones **1** is shown in Fig. 4. These reactions were performed using 18-crown-6/^*t*^BuOK (3.0 equiv)/HCF_3_ in THF at rt. Using THF as the solvent is important (see Table S3) and is discussed below. The strategy of tuning the Lewis acidity of the potassium-based counter-cations enabled the effective trifluoromethylation of enolizable ketones with fluoroform, although the need to use stoichiometric amounts of rather costly 18-crown-6 may limit the applicability of the method on a larger scale.

## Discussion

The reactive anionoid CF_3_ species in the mismatched Lewis acid-base adducts [K(polyethers)_n_][CF_3_] with diminished Lewis acidity of the K^+^ is rather stable, which is good agreement with the experimental observation in our previous report^38^. Namely, the sterically demanding and poorly electrophilic protonated ^*t*^BuP_4_ base, [H^*t*^BuP_4_]^+^, improves the reactivity and stability of the CF_3_^−^ for nucleophilic trifluoromethylation. This observation is in good agreement with the report by Prakash and co-workers that the anionoid CF_3_ species derived from ^*i*^Pr_3_SiCF_3_ in the presence of [K(18-crown-6)]^+^ is stable enough to be observed by NMR at −78 °C^44^. In spite of the apparent high degree of iconicity, the bonding between the coordinatively unsaturated and Lewis acidic K^+^ in [K(18-crown-6)]^+^ and the CF_3_ moiety certainly has a covalent component. Grushin and co-workers have reported the existence of the free or naked (uncoordinated) CF_3_^−^ anion with the [K(crypt-222)]^+^ counter-cation, in which the K^+^ is caged inside the 3-dimensional host^45^. This ionic complex has been characterized by a combination of methods, including X-ray diffraction, solution NMR, and reactivity toward electrophiles data, as well as labeling, acid-base, and DFT studies^45–47^.

**Figure 5:**
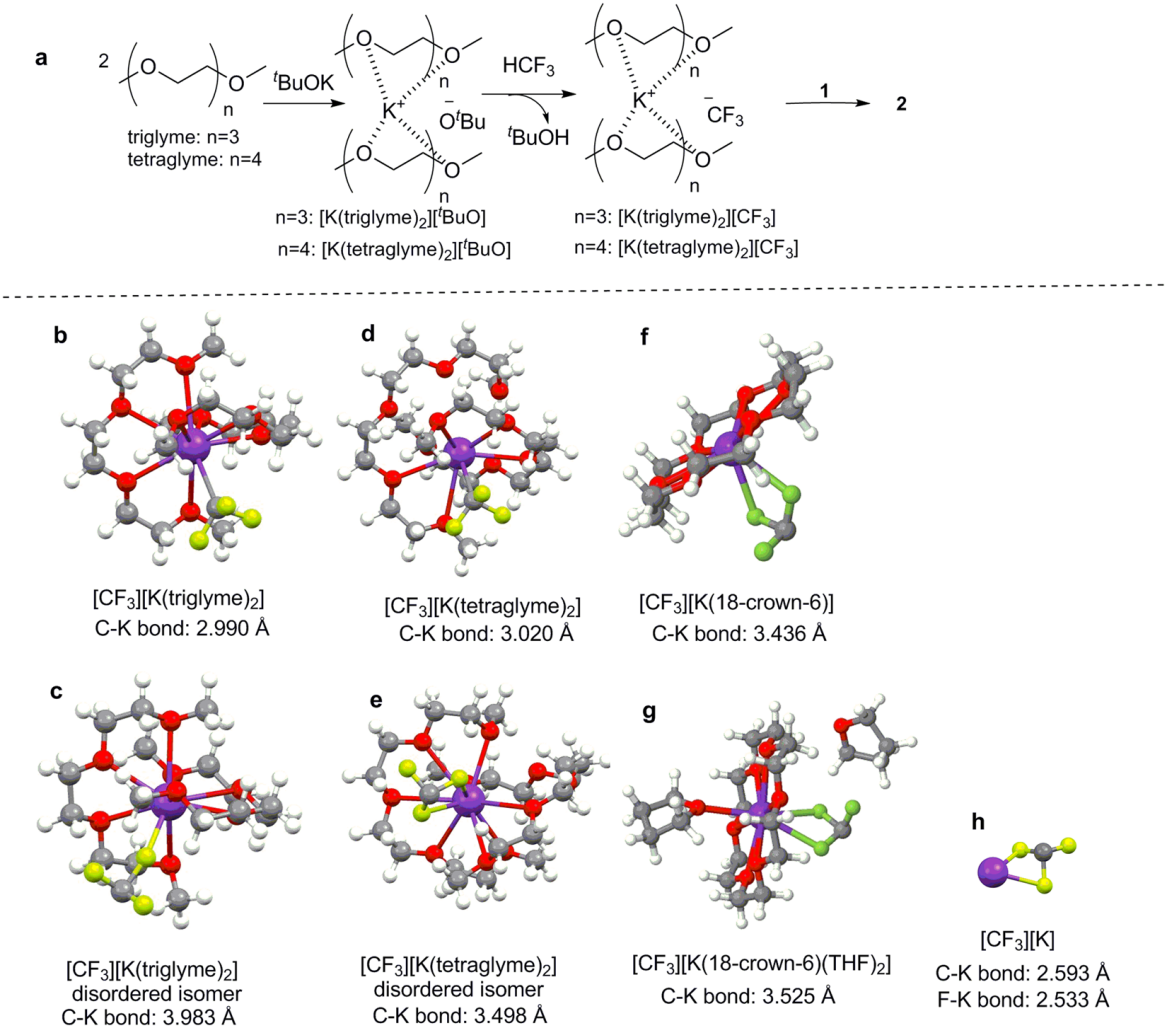
(**a**) Reaction mechanism for trifluoromethylation of **1** with HCF_3_/^*t*^BuOK in triglyme and tetraglyme. The optimized structures of (**b**) [*η*^1^-K(triglyme)_2_][CF_3_], (**c**) [*η*^1^-K(triglyme)_2_][CF_3_] disordered isomer, (**d**) [*η*^1^-K(tetraglyme)_2_][CF_3_], (**e**) [*η*^2^-K(tetraglyme)_2_][CF_3_] disordered isomer, (**f**) [K(18-crown-6)][CF_3_], (**g**) [K(18-crown-6)/THF][CF_3_] and [K][CF_3_] complexes obtained by B3LYP/6-311G** level DFT calculations.

As [K(polyether)_n_]CF_3_ intermediates are expected to be much more stable than KCF_3_ (see above), a reaction mechanism in glymes (triglyme or tetraglyme) is proposed as shown in Fig. 5a. First, two molecules of glymes coordinate to ^*t*^BuOK to form, reversibly, a 2:1 complex of [K(glyme)_2_][^*t*^BuO], followed by deprotonation of HCF_3_ with the ^*t*^BuO^−^ to furnish [K(glyme)_2_][CF_3_]. In this complex, the K^+^ is ligated by the glyme molecules, which reduces its Lewis acidity and, consequently, ability to decompose the CF_3_^−^. Similarly, if 18-crown-6 is used in place of the glyme, [K(18-crown-6)(^*t*^BuO)] is first formed, which deprotonates HCF_3_ to give [K(18-crown-6)(CF_3_)].

The structures of [K(triglyme)_2_][CF_3_] and [K(tetraglyme)_2_][CF_3_] were studied by DFT calculations^48,49^ using reported X-ray structural data for [K(triglyme)_2_]^+^^50^ and [K(tetraglyme)_2_]^+^ ^51^. The four selected minima identified (Fig. 5; see also the Supplementary Information) display coordination of the CF_3_ to the glyme-ligated K^+^ through the C or F atoms. This is also the case with the computed structures of KCF_3_ and [K(18-crown-6)(CF_3_)], in which K-F contacts were found. A deviation from the tetrahedral geometry is observed in all of the computed structures, featuring longer C-F bonds and distorted F-C-F angles. Without glyme ligands, optimized KCF_3_ displayed coordination via two of the three F atoms and an overall tighter bonding, as follows from the bond distances presented in Fig. 5. Naturally, the less Lewis acidic K^+^ interacts with CF_3_^−^ more weakly, which not only inhibits the undesired formation of KF and CF_2_, but also enhances the nucleophilicity of the anionoid CF_3_ species toward the organic substrate.

With regard to the beneficial effect of THF in the trifluoromethylation of enolizable ketones with HCF_3_ in the presence of 18-crown-6 (Fig. 4), we optimized the structure of [K(18-crown-6)(CF_3_)] in THF, using the X-data for [K(18-crown-6)(^*t*^BuO)]^48,49,52,53^. This structure [*η*^2^-K(18-crown-6)(CF_3_)] (Fig. 5f) also showed the coordination of the CF_3_ to the K center via two of the three fluorine atoms. Also, using the X-ray data for [K(18-crown-6)(THF)]^+^ ^54^, the structure of [K(18-crown-6)(THF)(CF_3_)] was computed (Fig. 5g)^48,49,53^. The binding energies (*E*_bind_) for [K(18-crown-6)(^*t*^BuO)] and [K(18-crown-6)(CF_3_)] in THF were computed at −26.7 and −24.1 kcal/mol, respectively^49,53^. For [K(18-crown-6)(THF)(^*t*^BuO)] and [K(18-crown-6)(THF)(CF_3_)], also in THF, the K-O and K-F interactions were weaker, according to the computed *E*_bind_ values of −19.3 and −20.1 kcal/mol, respectively^49,53^. We therefore conclude that THF is also capable of serving as a ligand to the potassium in the reaction, thereby additionally diminishing the Lewis acidity of the cation and consequently enhancing both the reactivity of the anionoid CF_3_ intermediate and its stability toward fluoride elimination

In summary, we have developed an advantageous, simple and high-yielding method to trifluoromethylation of ketones, chalcones and aldehydes to the corresponding trifluoromethyl carbinols with HCF_3_ and a potassium base in the presence of glymes and/or 18-crown-6. The beneficial event of the polyethers deals with their coordination to the K^+^, rendering it less prone to fluoride abstraction from the reactive anionoid CF_3_ intermediate. Our data provide complementary evidence for enhanced stability of CF_3_^−^ toward fluoride elimination and formation of difluorocarbene (:CF_2_), which is strongly induced by metal-fluorine interactions.

## Methods

### Trifluoromethylation of acyclic diaryl ketones 1a–1h, cyclic diaryl ketones 1o, 1p and bulky aliphatic-substituted ketones 1q, 1r by using monoglyme or triglyme as solvent in Table 1b (see Supplementary Information, the general synthetic procedure A and B)

The solution of ^*t*^BuOK (45 mg, 0.4 mmol) in dry monoglyme or triglyme (0.5 mL), was cooled in liquid nitrogen followed by adding carbonyl compounds (diaryl ketones **1a**–**1h**, cyclic diaryl ketones **1o**, **1p** and bulky aliphatic-substituted ketones **1q**, **1r**, 0.2 mmol) under argon atmosphere. After being charged with HCF_3_ (1.0 equiv or excess) by cooling at the same temperature under vacuum, the resulting mixture was allowed to warm to room temperature. Then the reaction mixture was stirred at rt for 6 h monitored by TLC, quenched by addition of sat. NH_4_Cl aq., extracted with Et_2_O, dried over with Na_2_SO_4_ and then concentrated in *vacuo*. The residue was purified by column chromatography on silica gel (*n*-hexane/ethyl acetate) to give corresponding α-trifluoromethyl alcohols **2a**–**2h**, **2o**–**2r** in good to high yields.

### Trifluoromethylation of nitro group substituted diaryl ketones 1i, heteroaryl groups substituted ketones 1j–1n and chalcones 1s–w, aryl aldehydes 1x–1z and 1aa-1ee by using triglyme as solvent in Table 1b (see Supplementary Information, the general synthetic procedure C and D)

The solution of ^*t*^BuOK (45 mg, 0.4 mmol) or KHMDS (80 mg, 0.4 mmol) in dry triglyme (0.5 mL) was charged with fluoroform by cooling in liquid nitrogen under vacuum. After being warmed to −40 °C, a solution of carbonyl compounds (0.20 mol) in triglyme (0.5 mL) was added slowly (over 5 min) by syringe. Then the reaction mixture was stirred at the same temperature for 12 h, quenched by addition of sat. NH_4_Cl aq., extracted with Et_2_O, dried over with Na_2_SO_4_ and then concentrated in *vacuo*. The residue was purified by column chromatography on silica gel (n-hexane/ethyl acetate) to give corresponding α-trifluoromethyl alcohols **2i**–**n** and **2s**–**2z** and **2aa**–**2ee** in good yields.

### Trifluoromethylation of enolizable ketones 1ff-1kk in the presence of 18-crown-6 in Fig. 4 (see Supplementary Information, the general synthetic procedure E)

The solution of ^*t*^BuOK (67 mg, 0.6 mmol), 18-crown-6 (159 mg, 0.6 mmol) in THF (2.0 mL) was cooled in liquid nitrogen followed by adding carbonyl compounds (enolizable ketones **1ff**–**1kk, 0.2 mmol**) under argon atmosphere. Then the resulting mixture was charged with HCF_3_ by cooling at the same temperature under vacuum. Then the solution was allowed to warm to room temperature. After being stirred for 6–12 h monitoring by TLC upon the completion of the reaction, the resulting mixture was quenched with sat. NH_4_Cl aq. extracted with Et_2_O, dried over with Na_2_SO_4_ and then concentrated in *vacuo*. The residue was purified by column chromatography on silica gel (n-hexane/ethyl acetate) to give corresponding α-trifluoromethyl alcohols **2ff**–**2kk** in good yields.

## Electronic supplementary material

Supplementary Information
