# Supplementary material for: Direct nucleophilic trifluoromethylation of carbonyl compounds by potent greenhouse gas, fluoroform: Improving the reactivity of anionoid trifluoromethyl species in glymes
Source: Sci Rep. 2018 Jul 31;8:11501. doi: 10.1038/s41598-018-29748-1 (PMC6068191; doi:10.1038/s41598-018-29748-1)
Supplement: Supplementary file 1 — Supplementary Information [file 41598_2018_29748_MOESM1_ESM.docx]

**Electronic Supplementary Information**

Direct nucleophilic trifluoromethylation of carbonyl compounds by potent greenhouse gas, fluoroform: Improving the reactivity of anionoid trifluoromethyl species in glymes

Takuya Saito,^^[[1]](#footnote-1)^^ Jiandong Wang,^1^ Etsuko Tokunaga,^1^ Seiji Tsuzuki^[[2]](#footnote-2)^ and Norio Shibata^1,^^[[3]](#footnote-3)*^

Contents

[1. Table S1: The coordinated situation between K^+^ and polyethers S1](#_Toc505694941)

[2. Table S2: Optimization of trifluoromethylation of chalcones S2](#_Toc505694942)

[3. Table S3: Optimization of trifluoromethylation of aryl aldehydes S2](#_Toc505694943)

[4. Table S4: Optimization of trifluoromethylation of enolizable ketones S3](#_Toc505694944)

[5. General procedures for trifluoromethylation of carbonyl compounds S4](#_Toc505694945)

[6. Copies of ^1^H, ^13^Cand ^19^F NMR spectra of new compounds 2g, 2k, 2m, 2n, 2v S17](#_Toc505694946)

[7. Copies of ^1^H and ^19^F NMR spectra for known compounds 2a-2f, 2h-2j, 2l, 2o-2u, 2w-2z, 2aa-2kk S25](#_Toc505694947)

[8. Computational methods S57](#_Toc505694948)

**General information:**

All reactions were performed in oven-dried glassware under positive pressure of nitrogen unless mentioned otherwise. Solvents were transferred via syringe and were introduced into reaction vessels though a rubber septum. All reactions were monitored by thin-layer chromatography (TLC) carried out on 0.25 mm Merck silica gel (60-F254). The TLC plates were visualized with UV light and KMnO_4_ in water/heat. Column chromatography was carried out on columns packed with silica gel (60N spherical neutral size 63-210 μm). The ^1^H-NMR (300 MHz), ^19^F-NMR (282 MHz), ^13^C-NMR (125 MHz) spectra for solution in CDCl_3_ were recorded on a Buruker Avance 500 and a Varian Mercury 300. Chemical shifts (δ) are expressed in ppm downfield from internal TMS (δ = 0.00) or C_6_F_6_ [δ = -162.2 (CDCl_3_)] as an internal standard. Mass spectra were recorded on a SHIMADZU GCMS-QP5050A (EI-MS) and SHIMAZU LCMS-2020 (ESI-MS). Melting points were recorded on a BÜCHI Melting Point M-565. Infrared spectra were recorded on a JASCO FT/IR-200 spectrometer

**Materials**:

Super dehydrated solvents such as tetrahydrofuran and toluene (water max 0.001%) were purchased from Wako Pure Chemical Industries, Ltd. and used under argon atmosphere. Monoglyme were purchased from Nacalai Tesque, Inc. and used in glove box without further purification. Diglyme, triglyme, tetraglyme and KHMDS were purchased from Aldrich Inc and used in glove box without further purification. *^t^*BuOK was purchased from Tokyo Chemical Industry Co., Ltd., and used in glove box without further purification.

# **1. Table S1: The coordinated situation between K^+^ and polyethers**

| Entries | *^t^*BuOK (equiv) | Additive | equiv | yield (%)^a^ |
| --- | --- | --- | --- | --- |
| 1 | 2.0 | - |  | 32 |
| 2 | 2.0 | monoglyme | 2.0 | 32 |
| 3 | 2.0 | monoglyme | 4.0 | 36 |
| 4 | 2.0 | monoglyme | 6.0 | 56 |
| 5 | 2.0 | monoglyme | 8.0 | 57 |
| 6 | 2.0 | monoglyme | 10.0 | 61 |
| 7 | 2.0 | diglyme | 2.0 | 59 |
| 8 | 2.0 | diglyme | 4.0 | 63 |
| 9 | 2.0 | diglyme | 6.0 | 86 |
| 10 | 2.0 | diglyme | 8.0 | 90 |
| 11 | 2.0 | diglyme | 10 | 94 |
| 12 | 2.0 | triglyme | 1.0 | 54 |
| 13 | 2.0 | triglyme | 2.0 | 74 |
| 14 | 2.0 | triglyme | 3.0 | 85 |
| 15 | 2.0 | triglyme | 4.0 | >99 |
| 16 | 2.0 | triglyme | 6.0 | >99 |
| 17 | 2.0 | triglyme | 8.0 | >99 |
| 18 | 2.0 | tetraglyme | 2.0 | >99 |
| 19 | 2.0 | tetraglyme | 4.0 | >99 |
| 20 | 2.0 | tetraglyme | 6.0 | >99 |
| 21 | 2.0 | tetraglyme | 8.0 | >99 |
| 22 | 1.0 | --- | --- | 21 |
| 23 | 1.0 | triglyme | 1.0 | 38 |
| 24 | 1.0 | triglyme | 2.0 | 58 |
| 25 | 1.0 | triglyme | 3.0 | 60 |
| 26 | 1.0 | triglyme | 4.0 | 61 |
| 27 | 1.0 | tetraglyme | 0.5 | 34 |
| 28 | 1.0 | tetraglyme | 1.0 | 57 |
| 29 | 1.0 | tetraglyme | 2.0 | 72 |
| 30 | 1.0 | tetraglyme | 3.0 | 71 |
| ^a^ ^19^F NMR yields with PhCF_3_ as an internal standard. | | | | |

**2. Table S2: Optimization of trifluoromethylation of chalcones.**

| entries | base | temp (°C) | yield (%)^a^ |
| --- | --- | --- | --- |
| 1^b^ | *^t^*BuOK | rt | 13 |
| 2^b^ | *^t^*BuOK | −10 | 14 |
| 3^c^ | *^t^*BuOK | −40 | 35 |
| 4^c^ | KHMDS | rt | 67 |
| 5^c^ | KHMDS | −40 | 88 |
| ^a 19^F NMR yields with PhCF_3_ as an internal standard; ^b^ Chalcones **1s** was added in one portion as solid state; ^c^ Chalcones **1s** in triglyme (0.5 mL) was added slowly to the solution of potassium base (2.0 equiv) and CF_3_H (excess) in triglyme (0.5 mL). | | | |

# **3. Table S3: Optimization of trifluoromethylation of aryl aldehydes**

| Entries | Base | temp (°C) | yield (%)^a^ |
| --- | --- | --- | --- |
| 1 | *^t^*BuOK | rt | 7 |
| 2 | *^t^*BuOK | 0 | 21 |
| 3 | *^t^*BuOK | –10 | 35 |
| 4 | *^t^*BuOK | –20 | 57 |
| 5 | *^t^*BuOK | –40 | 64 |
| 6^b^ | *^t^*BuOK | –40 | 66 |
| 7^b^ | KHMDS | –40 | 20 |
| 8^b, c^ | *^t^*BuOK | –40 | 25 |
| ^a 19^F NMR yields with PhCF_3_ as an internal standard; ^b^ Aryl aldehydes **1x** in triglyme (0.5 mL) was added slowly to the solution of potassium base (2.0 equiv) and CF_3_H (excess) in triglyme (0.5 mL); ^c^ THF was used as solvent instead of triglyme | | | |

# **4. Table S4: Optimization of trifluoromethylation of enolizable ketones**

| Entries | Solvent | Concentration | Base (equiv) | additive (equiv) | yield (%)^a^ |
| --- | --- | --- | --- | --- | --- |
| 1 | triglyme | 0.4 M | *t*BuOK (2.0) | --- | 6 |
| 2^b^ | triglyme | 0.4 M | KHMDS (2.0) | --- | NR |
| 3^b^ | triglyme | 0.4 M | *t*BuOK (2.0) | --- | NR |
| 4 | triglyme | 0.2 M | *t*BuOK (2.0) | --- | 7 |
| 5 | triglyme | 0.1 M | *t*BuOK (2.0) | --- | 14 |
| 6 | triglyme | 0.05 M | *t*BuOK (2.0) | --- | 15 |
| 7 | triglyme | 0.1 M | *t*BuOK (3.0) | --- | 18 |
| 8 | tetraglyme | 0.4 M | *t*BuOK (2.0) | --- | 14 |
| 9 | tetraglyme | 0.2 M | *t*BuOK (2.0) | --- | 24 |
| 10 | tetraglyme | 0.1 M | *t*BuOK (2.0) | --- | 54 |
| 11 | tetraglyme | 0.05 M | *t*BuOK (2.0) | --- | 54 |
| 12 | tetraglyme | 0.1 M | *t*BuOK (3.0) | --- | 59 |
| 13 | tetraglyme | 0.1 M | *t*BuOK (4.0) | --- | 59 |
| 14 | toluene | 0.1 M | *t*BuOK (2.0) | 18-crown-6 (2.0 equiv) | 10 |
| 15 | toluene | 0.1 M | *t*BuOK (1.0) | 18-crown-6 (1.0 equiv) | 7 |
| 16 | toluene | 0.1 M | *t*BuOK (3.0) | 18-crown-6 (1.0 equiv) | 10 |
| 17 | toluene | 0.1 M | *t*BuOK (1.0) | 18-crown-6 (3.0 equiv) | 19 |
| 18 | THF | 0.1 M | *t*BuOK (3.0) | 18-crown-6 (3.0 equiv) | 85 |
| 19 | THF | 0.1 M | *t*BuOK (2.0) | 18-crown-6 (2.0 equiv) | 25 |
| 20 | toluene | 0.1 M | *t*BuOK (3.0) | cryptand (3.0 equiv) | 95 |
| 21 | THF | 0.1 M | *t*BuOK (3.0) | cryptand (3.0 equiv) | 96 |
| ^a^ The yields were determined by ^19^F NMR with PhCF_3_ as an internal standard;  ^b^ The reaction was conducted at –40 °C. | | | | | |

# **5. General procedures for trifluoromethylation of carbonyl compounds**

**5.1 General procedure (A):**Trifluoromethylation of acyclic diaryl ketones **1a-1h**, cyclic diaryl ketones **1o**, **1p** and bulky aliphatic-substituted ketones **1q**, **1r** by using monoglyme as solvent in Scheme 2.

In glove box, a dried 10 mL vessel was successively charged with *^t^*BuOK (45 mg, 0.4 mmol) and dry monoglyme (0.5 mL), and was sealed by a rubber septum and brought to bench. Subsequently, the solution was cooled in liquid nitrogen followed by adding carbonyl compounds (diaryl ketones **1a**-**1h**, cyclic diaryl ketones **1o**, **1p** and bulky aliphatic-substituted ketones **1q**, **1r**) under argon atmosphere. After being charged with HCF_3_ by cooling at the same temperature under vacuum, the resulting mixture was allowed to warm to room temperature. Then the reaction mixture was stirred at rt for 6 h monitored by TLC, quenched by addition of sat. NH_4_Cl aq., extracted with Et_2_O, dried over with Na_2_SO_4_ and then concentrated in *vacuo*. The residue was purified by column chromatography on silica gel (*n*-hexane/ethyl acetate) to give corresponding α-trifluoromethyl alcohols **2a-2h,**.**2o-2r** in good to high yields.

**5.2 General procedure (B):** Trifluoromethylation of diaryl ketones **1a**-**1h**, cyclic diaryl ketones **1o**, **1p** and bulky aliphatic-substituted ketones **1q**, **1r** by using triglyme as solvent in Scheme 2.

In glove box, a dried 10 mL vessel was successively charged with *^t^*BuOK (45 mg, 0.4 mmol) and dry triglyme (0.5 mL), and was sealed by a rubber septum and brought to bench. Subsequently, the solution was cooled in liquid nitrogen followed by adding carbonyl compounds (diaryl ketones **1a**-**1h**, cyclic diaryl ketones **1o**, **1p** and bulky aliphatic-substituted ketones **1q**, **1r**) under argon atmosphere. After being charged with HCF_3_ by cooling at the same temperature under vacuum, the resulting mixture was allowed to warm to room temperature. Then the reaction mixture was stirred at rt for 6 h monitored by TLC, quenched by addition of sat. NH_4_Cl aq., extracted with Et_2_O, dried over with Na_2_SO_4_ and then concentrated in vacuo. The residue was purified by column chromatography on silica gel (n-hexane/ethyl acetate) to give corresponding α-trifluoromethyl alcohols **2a-2h,**.**2o-2r** in good to high yields.

**2,2,2-Trifluoro-1,1-diphenylethanol (2a)**

This compound has been previously prepared and characterized.^1^

2,2,2-Trifluoro-1,1-diphenylethanol **2a** was prepared following general procedure (A): the reaction of benzophenone **1a** (36 mg, 0.20 mmol) with HCF_3_ (excess) was carried out in the presence of *^t^*BuOK (45 mg, 0.4 mmol) in monoglyme (0.5 mL) at room temperature for 6 h. Isolated by column chromatography on silica gel (*n*-hexane/ethyl acetate = 95/5) to give **2a** (44 mg, 86% yield) as a colorless oil. Following general procedure (B): the reaction of benzophenone **1a** (36 mg, 0.20 mmol) with HCF_3_ (excess) was carried out in the presence of *^t^*BuOK (45 mg, 0.4 mmol) in triglyme (0.5 mL) at room temperature for 6 h. Isolated by column chromatography on silica gel (*n*-hexane/ethyl acetate = 95/5) to give **2a** (48 mg, 95% yield) as a colorless oil. ^1^H NMR (CDCl_3_, 300 MHz) δ 2.88 (s, 1H), 7.33–7.40 (m, 6H), 7.48–7.51 (m, 4H); ^19^F NMR (CDCl_3_, 282 MHz) δ −74.8 (s, 3F); MS (ESI, *m/z*) 251 [M−H]^−^

**2,2,2-Trifluoro-1-(4-methoxyphenyl)-1-phenylethanol (2b)**

This compound has been previously prepared and characterized.^1^

2,2,2-Trifluoro-1-(4-methoxyphenyl)-1-phenylethanol **2b** was prepared following general procedure (A): the reaction of 4-methoxybenzophenone **1b** (42 mg, 0.20 mmol).with HCF_3_ (excess) was carried out in the precence of *^t^*BuOK (45 mg, 0.4 mmol) in monoglyme (0.5 mL) at room temperature for 6 h. Isolated by column chromatography on silica gel (*n*-hexane/ethyl acetate = 98/2) to give **2b** (44 mg, 77% yield) as a colorless oil. Following general procedure (B): the reaction of 4-methoxybenzophenone **1b** (42 mg, 0.20 mmol).with HCF_3_ (excess) was carried out in the presence of *^t^*BuOK (45 mg, 0.4 mmol) in triglyme (0.5 mL) at room temperature for 6 h. Isolated by column chromatography on silica gel (*n*-hexane/ethyl acetate = 95/5) to give **2b** (54 mg, 95% yield) as a colorless oil. ^1^H NMR (CDCl_3_, 300 MHz) δ 2.86 (s, 1H), 3.80 (s, 3H), 6.87 (d, *J* = 9.0 Hz, 2H), 7.34–7.41 (m, 5H), 7.48–7.51 (m, 2H); ^19^F NMR (CDCl_3_, 282 MHz) δ −75.0 (s, 3F); MS (ESI, *m/z*) 281 [M−H]^−^

**2,2,2-Trifluoro-1-phenyl-1-*p*-tolylethanol (2c)**

This compound has been previously prepared and characterized.^1^

2,2,2-Trifluoro-1-phenyl-1-*p*-tolylethanol **2c** was prepared following general procedure (A): the reaction of using 4-methylbenzophenone **1c** (39 mg, 0.20 mmol) with HCF_3_ (excess) was carried out in the presence of *^t^*BuOK (45 mg, 0.4 mmol) in monoglyme (0.5 mL) at room temperature for 6 h. Isolated by column chromatography on silica gel (*n*-hexane/ethyl acetate = 98/2) to give **2c** (43 mg, 80% yield) as a colorless oil. Following general procedure (B): the reaction of 4-methylbenzophenone **1c** (40 mg, 0.20 mmol) with HCF_3_ (excess) was carried out in the presence of *^t^*BuOK (45 mg, 0.4 mmol) in triglyme (0.5 mL) at room temperature for 6 h. Isolated by column chromatography on silica gel (*n*-hexane/ethyl acetate = 98/2) to give **2c** (50 mg, 94% yield) as a colorless oil. ^1^H NMR (CDCl_3_, 300 MHz) δ 2.35 (s, 3H), 2.85 (s, 1H), 7.17 (d, *J* = 8.1 Hz, 2H), 7.34–7.38 (m, 5H), 7.47–7.50 (m, 2H); ^19^F NMR (CDCl_3_, 282 MHz) δ −74.9 (s, 3F); MS (ESI, *m/z*) 265 [M−H]^−^

**1,1-Bis(4-chlorophenyl)-2,2,2-trifluoroethanol (2d)**

This compound has been previously prepared and characterized.^1^

1,1-Bis(4-chlorophenyl)-2,2,2-trifluoroethanol **2d** was prepared following general procedure (A): the reaction of 4, 4'-dichorobenzophenone **1d** (50 mg, 0.20 mmol) with HCF_3_ (excess) was carried out in the presence of *^t^*BuOK (45 mg, 0.4 mmol) in monoglyme (0.5 mL) at room temperature for 6 h. Isolated by column chromatography on silica gel (*n*-hexane/ethyl acetate = 95/5) to give **2d** (60 mg, 93% yield)as a colorless oil. Following general procedure (B): the reaction of 4, 4'-dichorobenzophenone **1d** (50 mg, 0.20 mmol) with HCF_3_ (excess) was carried out in the presence of *^t^*BuOK (45 mg, 0.4 mmol) in triglyme (0.5 mL) at room temperature for 6 h. Isolated by column chromatography on silica gel (*n*-hexane/ethyl acetate = 95/5) to give **2d** (60 mg, 93% yield) as a colorless oil. ^1^H NMR (CDCl_3_, 300 MHz) δ 2.97 (s, 1H), 7.32–7.35 (m, 4H), 7.40 (d, *J* = 8.7 Hz, 4H); ^19^F NMR (CDCl_3_, 282 MHz) δ −75.1 (s, 3F); MS (ESI, *m/z*) 319 [M−H]^−^

**1-(3-Chlorophenyl)-2,2,2-trifluoro-1-phenylethanol (2e)**

This compound has been previously prepared and characterized.^1^

1-(3-Chlorophenyl)-2,2,2-trifluoro-1-phenylethanol **2e** was prepared following general procedure (A): the reaction of 3-chorobenzophenone **1e** (43 mg, 0.20 mmol) with HCF_3_ (excess) was carried out in the presence of *^t^*BuOK (45 mg, 0.4 mmol) in monoglyme (0.5 mL) at room temperature for 6 h. Isolated by column chromatography on silica gel (*n*-hexane/ethyl acetate = 95/5) to give (51 mg, 89% yield) as a colorless oil. Following general procedure (B): the reaction of 3-chorobenzophenone **1e** (43 mg, 0.20 mmol) with HCF_3_ (excess) was carried out in the presence of *^t^*BuOK (45 mg, 0.4 mmol) in triglyme (0.5 mL) at room temperature for 6 h. Isolated by column chromatography on silica gel (*n*-hexane/ethyl acetate = 95/5) to give ~~(~~53 mg, 92% yield) as a colorless oil. ^1^H NMR (CDCl_3_, 300 MHz) δ 2.93 (s, 1H), 7.28–7.40 (m, 6H), 7.47–7.50 (m, 2H), 7.53 (s, 1H); ^19^F NMR (CDCl_3_, 282 MHz) δ −74.8 (s, 3F); MS (ESI, *m/z*) 285 [M−H]^−^

**2,2,2-Trifluoro-1-(4-fluorophenyl)-1-phenylethanol (2f)**

This compound has been previously prepared and characterized.^2^

2,2,2-Trifluoro-1-(4-fluorophenyl)-1-phenylethanol **2f** was prepared following general procedure (A): the reaction (4-fluorophenyl)(phenyl)methanone **1f** (40 mg, 0.20 mmol) with HCF_3_ (excess) was carried out in the presence of *^t^*BuOK (45 mg, 0.4 mmol) in monoglyme (0.5 mL) at room temperature for 6 h. Isolated by column chromatography on silica gel (*n*-hexane/ethyl acetate = 95/5) to give **2f** (39 mg, 72% yield) as a colorless oil. Following general procedure (B): the reaction of (4-fluorophenyl)(phenyl)methanone **1f** (40 mg, 0.20 mmol) with HCF_3_ (excess) was carried out in the presence of *^t^*BuOK (45 mg, 0.4 mmol) in triglyme (0.5 mL) at room temperature for 6 h. Isolated by column chromatography on silica gel (*n*-hexane/ethyl acetate = 95/5) to give **2f** (52 mg, 98% yield) as a colorless oil. ^1^H NMR (CDCl_3_, 300 MHz) δ 2.90 (s, 1H), 7.04 (t, *J* = 8.9 Hz, 2H), 7.36–7.39 (m, 3H), 7.44–7.49 (m, 4H); ^19^F NMR (CDCl_3_, 282 MHz) δ −133.67–−133.58 (m, 1F), −75.0 (s, 3F); MS (ESI, *m/z*) 269 [M−H]^−^

**1,1-Bis(4-bromophenyl)-2,2,2-trifluoroethanol (2g)**

1,1-Bis(4-bromophenyl)-2,2,2-trifluoroethanol **2g** was prepared following general procedure (A): the reaction 4, 4'-dibromobenzophenone **1g** (68 mg, 0.20 mmol) with HCF_3_ (excess) was carried out in the presence of *^t^*BuOK (45 mg, 0.4 mmol) in monoglyme (0.5 mL) at room temperature for 6 h. Isolated by column chromatography on silica gel (*n*-hexane/ethyl acetate = 95/5) to give **2g** (67 mg, 81% yield) as a colorless oil. Following general procedure (B): the reaction of 4, 4'-dibromobenzophenone **1g** (68 mg, 0.20 mmol) with HCF_3_ (excess) was carried out in the presence of *^t^*BuOK (45 mg, 0.4 mmol) in triglyme (0.5 mL) at room temperature for 6 h. Isolated by column chromatography on silica gel (*n*-hexane/ethyl acetate = 95/5) to give **2g** (70 mg, 86% yield) as a yellow oil. ^1^H NMR (CDCl_3_, 300 MHz) δ 2.97 (s, 1H), 7.34 (d, *J* = 8.4 Hz, 4H), 7.50 (d, *J* = 8.7 Hz, 4H); ^13^C NMR (CDCl_3_, 125.8 MHz) δ 78.9 (q, *J* = 29.1 Hz), 123.3, 124.8 (q, *J* = 286.4 Hz), 129.1, 131.5, 137.8; ^19^F NMR (CDCl_3_, 282 MHz) δ −75.1 (s, 3F); IR (neat) 3543, 1909, 1590, 1490, 1590, 1490, 1401, 1283, 1265, 1171, 1076, 1011, 909, 819, 744, 722, 687 cm^−1^; MS (ESI, *m/z*) 407 [M−H]^−^; HRMS (ESI, m/z) [M−H]^−^ Calcd. for C_14_H_8_Br_2_F_3_O 406.8894, Found 406.8895.

**2,2,2-Trifluoro-1-(4-(trifluoromethyl)phenyl)-1-phenylethanol (2h)**

This compound has been previously prepared and characterized.^3^

2,2,2-Trifluoro-1-(4-(trifluoromethyl)phenyl)-1-phenylethanol **2h** was prepared following general procedure (A): the reaction of 4-trifluoromethybenzophenone **1h** (50 mg, 0.20 mmol) with HCF_3_ (excess) was carried out in the presence of *^t^*BuOK (45 mg, 0.4 mmol) in monoglyme (0.5 mL) at room temperature for 6 h. Isolated by column chromatography on silica gel (*n*-hexane/ethyl acetate = 95/5) to give (53 mg, 83% yield) as a colorless oil. Following general procedure (B): the reaction of 4-trifluoromethybenzophenone **1h** (50 mg, 0.20 mmol) with HCF_3_ (excess) was carried out in the presence of *^t^*BuOK (45 mg, 0.4 mmol) in triglyme (0.5 mL) at room temperature for 6 h. Isolated by column chromatography on silica gel (*n*-hexane/ethyl acetate = 95/5) to give **2h** (64 mg, 99% yield) as a colorless oil. ^1^H NMR (CDCl_3_, 300 MHz) δ 2.99 (s, 1H), 7.38–7.41 (m, 3H), 7.46–7.49 (m, 2H), 7.62 (s, 4H); ^19^F NMR (CDCl_3_, 282 MHz) δ −74.8 (s, 3F), −63.3 (s, 3F); MS (ESI, *m/z*) 319 [M−H]^−^

**9-(Trifluoromethyl)-9*H*-fluoren-9-ol (2o)**

This compound has been previously prepared and characterized.^3^

9-(Trifluoromethyl)-9*H*-fluoren-9-ol **2o** was prepared following general procedure (A): the reaction of fluorenone **1o** (36 mg, 0.20 mmol) with HCF_3_ (excess) was carried out in the presence of *^t^*BuOK (45 mg, 0.4 mmol) in monoglyme (0.5 mL) at room temperature for 6 h. Isolated by column chromatography on silica gel (*n*-hexane/ethyl acetate = 90/10) to give **2o** (33 mg, 66% yield) as a white solid. Following general procedure (B): the reaction of fluorenone **1o** (36 mg, 0.20 mmol) with HCF_3_ (excess) was carried out in the presence of *^t^*BuOK (45 mg, 0.4 mmol) in triglyme (0.5 mL) at room temperature for 6 h. Isolated by column chromatography on silica gel (*n*-hexane/ethyl acetate = 90/10) to give **2o** (46 mg, 91% yield) as a white solid. ^1^H NMR (CDCl_3_, 300 MHz) δ 2.70 (s, 1H), 7.36 (td, *J* = 7.5, 0.9 Hz 2H), 7.48 (td, *J* = 7.5, 0.9 Hz 2H), 7.66–7.72 (m, 4H); ^19^F NMR (CDCl_3_, 282 MHz) δ −79.1 (s, 3F); MS (ESI, *m/z*) 249 [M−H]^−^.

**9-(Trifluoromethyl)-9*H*-xanthen-9-ol (2p)**

This compound has been previously prepared and characterized.^2^

9-(Trifluoromethyl)-9*H*-xanthen-9-ol **2p** was prepared following general procedure (A): the reaction of xanthone **1p** (39 mg, 0.20 mmol) with HCF_3_ (excess) was carried out in the presence of *^t^*BuOK (45 mg, 0.4 mmol) in monoglyme (0.5 mL) at room temperature for 6 h. Isolated by column chromatography on silica gel (*n*-hexane/ethyl acetate = 95/5) to give **2p** (27 mg, 50% yield) as a white solid. Following general procedure (B): the reaction of xanthone **1p** (39 mg, 0.20 mmol) with HCF_3_ (excess) was carried out in the presence of *^t^*BuOK (45 mg, 0.4 mmol) in triglyme (0.5 mL) at room temperature for 6 h. Isolated by column chromatography on silica gel (*n*-hexane/ethyl acetate = 90/10) to give **2p** (51 mg, 95% yield) as a white solid. ^1^H NMR (CDCl_3_, 300 MHz) δ 2.87 (s, 1H), 7.21–7.28 (m, 4H), 7.43–7.46 (m, 2H), 7.85 (d, *J* = 7.8 Hz, 2H); ^19^F NMR (CDCl_3_, 282 MHz) δ −81.9 (s, 3F); MS (ESI, *m/z*) 265 [M−H]^−^

**2-(Trifluoromethyl)-2-adamantanol (2q)**

This compound has been previously prepared and characterized.^1^

2-(Trifluoromethyl)-2-adamantanol **2q** was prepared following general procedure (A): the reaction of 2-adamantanone **1q** (30 mg, 0.20 mmol) with HCF_3_ (excess) was carried out in the presence of *^t^*BuOK (45 mg, 0.4 mmol) in monoglyme (0.5 mL) at room temperature for 6 h. Isolated by column chromatography on silica gel (*n*-hexane/ethyl acetate = 98/2) to give give **2q** (37 mg, 84% yield) as a white solid. Following general procedure (B): the reaction of 2-adamantanone **1q** (30 mg, 0.20 mmol) with HCF_3_ (excess) was carried out in the presence of *^t^*BuOK (45 mg, 0.4 mmol) in triglyme (0.5 mL) at room temperature for 6 h. Isolated by column chromatography on silica gel (*n*-hexane/ethyl acetate = 98/2) to give **2q** (38 mg, 87% yield) as a white solid. ^1^H NMR (CDCl_3_, 300 MHz) δ 1.61 (d, *J* = 13.2 Hz, 2H), 1.75–1.79 (m, 4H), 1.84–1.91 (m, 3H), 2.08–2.12 (m, 4H), 2.25 (d, *J* = 12.6 Hz, 2H); ^19^F NMR (CDCl_3_, 282 MHz) δ −76.1 (s, 3F); MS (ESI, *m/z*) 219 [M−H]^−^

**1,1,1-Trifluoro-3,3-dimethyl-2-phenylbutan-2-ol (2r)**

This compound has been previously prepared and characterized.^2^

1,1,1-Trifluoro-3,3-dimethyl-2-phenylbutan-2-ol **2r** was prepared following general procedure (A): the reaction of 2,2-dimethylpropiophenone **1r** (32 mg, 0.20 mmol). with HCF_3_ (excess) was carried out in the presence of *^t^*BuOK (45 mg, 0.4 mmol) in monoglyme (0.5 mL) at room temperature for 6 h. Isolated by column chromatography on silica gel (*n*-hexane/ethyl acetate = 98/2) to give **2r** (35 mg, 76% yield) as a colorless oil. Following general procedure (B): the reaction of 2,2-dimethylpropiophenone **1r** (32 mg, 0.20 mmol) with HCF_3_ (excess) was carried out in the presence of *^t^*BuOK (45 mg, 0.4 mmol) in triglyme (0.5 mL) at room temperature for 6 h. Isolated by column chromatography on silica gel (*n*-hexane/ethyl acetate = 95/5) to give **2r** (37 mg, 79% yield) as a colorless oil. ^1^H NMR (CDCl_3_, 300 MHz) δ 1.04 (s, 9H), 2.51 (s, 1H), 7.35–7.39 (m, 3H), 7.57 (s, 2H); ^19^F NMR (CDCl_3_, 282 MHz) δ −68.6 (s, 3F); MS (ESI, *m/z*) 231 [M−H]^−^

**5.3 General procedure (C)**: Trifluoromethylation of nitro group substituted diaryl ketones **1i**, heteroaryl groups substituted ketones **1j**-**1n** and chalcones **1s**-**w** by using triglyme as solvent in Scheme 2.

In glove box, a dried 10 mL vessel was successively charged with KHMDS (80 mg, 0.4 mmol) and dry triglyme (0.5 mL), and was sealed by a rubber septum and brought to bench. After being charged with fluoroform by cooling in liquid nitrogen under vacuum, the resulting mixture was warmed to −40 ^o^C and a solution of carbonyl compounds **1i**-**n** and **1s-w** (0.20 mol) in triglyme (0.5 mL) was added slowly (over 5 min) by syringe. Then the reaction mixture was stirred at the same temperature for 12 h, quenched by addition of sat. NH_4_Cl aq., extracted with Et_2_O, dried over with Na_2_SO_4_ and then concentrated *in vacuo.* The residue was purified by column chromatography on silica gel (n-hexane/ethyl acetate) to give corresponding α-trifluoromethyl alcohol **2i**-**n** and **2s-w** in good to high yields.

**2,2,2-Trifluoro-1-(4-nitrophenyl)-1-phenylethan-1-ol (2i)**

This compound has been previously prepared and characterized.^1^

2,2,2-Trifluoro-1-(4-nitrophenyl)-1-phenylethan-1-ol **2i** was prepared following general procedure (C): 4-nitrobenzophenone **1i** (46mg, 0.20 mmol) in triglyme (0.5 mL) was added slowly to the solution of KHMDS (80 mg, 0.4 mmol) and HCF_3_ (excess) in triglyme (0.5 mL) at –40 ^o^C. And the resulting mixture was stirred at the same temperature for 12 h. Isolated by column chromatography on silica gel (*n*-hexane/ethyl acetate = 95/5) to **2i** (46 mg, 77% yield) as a white solid. ^1^H NMR (CDCl_3_, 300 MHz) δ 3.11 (s, 1H), 7.39–7.42 (m, 3H), 7.46–7.48 (m, 2H), 7.70 (d, *J* = 8.4 Hz, 2H), 8.21 (d, *J* = 9.0 Hz, 2H); ^19^F NMR (CDCl_3_, 282 MHz) δ −74.7 (s, 3F); MS (ESI, *m/z*) 296[M−H]^−^

**2,2,2-Trifluoro-1-phenyl-1-(thiophen-2-yl)ethan-1-ol (2j)**

This compound has been previously prepared and characterized.^4^

2,2,2-Trifluoro-1-phenyl-1-(thiophen-2-yl)ethan-1-ol **2j** was prepared following general procedure (C): 2-benzoylthiophene **1j** (38 mg, 0.20 mmol) in triglyme (0.5 mL) was added slowly to the solution of KHMDS (80 mg, 0.4 mmol) and HCF_3_ (excess) in triglyme (0.5 mL) at –40 ^o^C. And the resulting mixture was stirred at the same temperature for 12 h. Isolated by column chromatography on silica gel (*n*-hexane/ethyl acetate = 95/5) to **2j** (42 mg, 81% yield) as a colorless oil. ^1^H NMR (CDCl_3_, 300 MHz) δ 3.11 (s, 1H), 7.02 (dd, *J* = 5.1, 3.9 Hz, 1H), 7.21–7.23 (m, 1H), 7.35–7.40 (m, 4H), 7.59–7.62 (m, 2H); ^19^F NMR (CDCl_3_, 282 MHz) δ −76.8 (s, 3F); MS (ESI, *m/z*) 257 [M−H]^−^

**2,2,2-Trifluoro-1-(furan-2-yl)-1-phenylethan-1-ol (2k)**

2,2,2-Trifluoro-1-(furan-2-yl)-1-phenylethan-1-ol **2k** was prepared following general procedure (C): 2-benzoylfuran **1k** (34 mg, 0.20 mmol) in triglyme (0.5 mL) was added slowly to the solution of KHMDS (80 mg, 0.4 mmol) and HCF_3_ (excess) in triglyme (0.5 mL) at –40 ^o^C. And the resulting mixture was stirred at the same temperature for 12 h. Isolated by column chromatography on silica gel (*n*-hexane/ethyl acetate = 95/5) to **2k** (36 mg, 74% yield) as a colorless oil. ^1^H NMR (CDCl_3_, 300 MHz) δ 3.20 (s, 1H), 6.42–6.43 (m, 1H), 6.50–6.51 (m, 1H), 7.39–7.41 (m, 3H), 7.45–7.46 (m, 1H), 7.54–7.57 (m, 2H); ^13^C NMR (CDCl_3_, 125.8 MHz) δ 76.1 (q, *J* = 30.0 Hz), 110.3 (m), 110.5, 124.2 (q, *J* = 284.2 Hz), 127.0, 128.2, 129.1, 135.5, 143.5, 150.6; ^19^F NMR (CDCl_3_, 282 MHz) δ −77.5 (s, 3F); IR (neat) 3542, 3066, 2930, 1707, 1499, 1452, 1354, 1267, 1227, 1173, 1025, 964, 913, 870, 761, 745, 724, 697, 659, 596, 508 cm^−1^; MS (ESI, *m/z*) 241 [M−H]^−^, HRMS (ESI, *m/z*) [M−H]^−^ Calcd. for C_12_H_8_F_3_O_2_ 241.0476 : Found 241.0477.

**2,2,2-Trifluoro-1-phenyl-1-(pyridin-2-yl)ethan-1-ol (2l)**

This compound has been previously prepared and characterized.^5^

2,2,2-Trifluoro-1-phenyl-1-(pyridin-2-yl)ethan-1-ol **2l** was prepared following general procedure (C): 2-benzoylpyridine **1l** (37 mg, 0.20 mmol).in triglyme (0.5 mL) was added slowly to the solution of KHMDS (80 mg, 0.4 mmol) and HCF_3_ (excess) in triglyme (0.5 mL) at –40 ^°^C. And the resulting mixture was stirred at the same temperature for 12 h. Isolated by column chromatography on silica gel (*n*-hexane/ethyl acetate = 95/5) **2l** (47 mg, 92% yield) as a white solid. ^1^H NMR (CDCl_3_, 300 MHz) δ 7.03 (s, 1H), 7.32–7.40 (m, 4H), 7.48 (d, *J* = 8.1 Hz, 1H), 7.65 (s, 1H), 7.67 (s, 1H), 7.73 (td, *J* = 7.8, 1.8 Hz, 1H), 8.59 (d, *J* = 4.8 Hz, 1H); ^19^F NMR (CDCl_3_, 282 MHz) δ −75.2 (s, 3F); MS (ESI, *m/z*) 252 [M−H]^−^

**2,2,2-Trifluoro-1-phenyl-1-(pyridin-3-yl)ethan-1-ol (2m)**

2,2,2-Trifluoro-1-phenyl-1-(pyridin-3-yl)ethan-1-ol **2m** was prepared following general procedure (C): 3-benzoylpyridine **1m** (37 mg, 0.20 mmol) in triglyme (0.5 mL) was added slowly to the solution of KHMDS (80 mg, 0.4 mmol) and HCF_3_ (excess) in triglyme (0.5 mL) at –40 ^°^C. And the resulting mixture was stirred at the same temperature for 12 h. Isolated by column chromatography on silica gel (*n*-hexane/ethyl acetate = 70/30) **2m** (44 mg, 87% yield) as a white solid. mp = 111–112 °C; ^1^H NMR (CDCl_3_, 300 MHz) δ 5.08 (s, 1H), 7.22–7.27 (m, 1H), 7.35–7.38 (m, 3H), 7.45–7.48 (m, 2H), 7.81 (d, *J* = 8.1 Hz, 1H), 8.37–8.38 (m, 1H), 8.49 (s, 1H); ^13^C NMR (CDCl_3_, 125.8 MHz) δ 78.1 (q, *J* = 29.1 Hz), 123.0, 125.1 (q, *J* = 286.9 Hz), 127.3, 128.4, 128.8, 135.7, 136.1, 138.8, 148.2, 148.5; ^19^F NMR (CDCl_3_, 282 MHz) δ −74.9 (s, 3F); IR (neat) 3079, 3030, 2749, 2605, 2096, 1597, 1582, 1483, 1449, 1425, 1267, 1194, 1149, 936, 922, 910, 762, 740, 699, 658 cm^−1^; mp: 111–112 °C; MS (ESI, *m/z*) 252 [M−H]^−^, HRMS (ESI, *m/z*) [M−H]^−^ Calcd. for C_13_H_9_F_3_NO 252.0636: Found 252.0641.

**2,2,2-Trifluoro-1-phenyl-1-(pyridin-4-yl)ethan-1-ol (2n)**

2,2,2-Trifluoro-1-phenyl-1-(pyridin-4-yl)ethan-1-ol **2n** was prepared following general procedure (C): 4-benzoylpyridine **1n** (37 mg, 0.20 mmol). in triglyme (0.5 mL) was added slowly to the solution of KHMDS (80 mg, 0.4 mmol) and HCF_3_ (excess) in triglyme (0.5 mL) at –40 ^o^C. And the resulting mixture was stirred at the same temperature for 12 h. Isolated by column chromatography on silica gel (*n*-hexane/ethyl acetate = 70/30) **2n** (45 mg, 89% yield) as a white solid. mp = 174–175 °C; ^1^H NMR (DMSO-*d*_6_, 300 MHz) δ 7.40–7.44 (m, 7H), 7.71 (s, 1H), 8.61–8.63 (m, 2H); ^13^C NMR (DMSO-*d*_6_, 125.8 MHz) δ 78.1 (q, *J* = 27.9 Hz), 121.9, 125.2 (q, *J* = 283.1 Hz), 127.1, 128.4, 128.7, 138.7, 148.5, 149.8; ^19^F NMR (DMSO-*d*_6_, 282 MHz) δ −74.8 (s, 3F); IR (neat) 3068, 2796, 1601, 1498, 1449, 1271, 1162, 1068, 1006, 948, 937, 814, 760, 739, 697, 673, 652 cm^−1^; MS (ESI, *m/z*) 252 [M−H]^−^, HRMS (ESI, *m/z*) [M−H]^−^ Calcd. for C_13_H_9_F_3_NO 252.0636: Found 252.0628.

**(*E*)-1,1,1-Trifluoro-2,4-diphenylbut-3-en-2-ol (2s)**

This compound has been previously prepared and characterized.^3^

(*E*)-1,1,1-Trifluoro-2,4-diphenylbut-3-en-2-ol **2s** was prepared following general procedure (C): chalcone **1s** (42 mg, 0.20 mmol) in triglyme (0.5 mL) was added slowly to the solution of KHMDS (80 mg, 0.4 mmol) and HCF_3_ (excess) in triglyme (0.5 mL) at –40 ^o^C. And the resulting mixture was stirred at the same temperature for 12 h. Isolated by column chromatography on silica gel (*n*-hexane/ethyl acetate = 95/5) **2s** (49 mg, 88% yield) as a colorless oil. ^1^H NMR (CDCl_3_, 300 MHz) δ 2.69 (s, 1H), 6.72 (d, *J* = 15.9 Hz, 1H), 6.89 (d, *J* = 15.9 Hz, 1H), 7.29–7.44 (m, 8H), 7.63–7.66 (m, 2H); ^19^F NMR (CDCl_3_, 282 MHz) δ −79.0 (m, 3F); MS (ESI, *m/z*) 277 [M−H]^−^

**(*E*)-1,1,1-Trifluoro-2-(4-methoxyphenyl)-4-phenylbut-3-en-2-ol (2t)**

This compound has been previously prepared and characterized.^3^

(*E*)-1,1,1-Trifluoro-2-(4-methoxyphenyl)-4-phenylbut-3-en-2-ol **2t** was prepared following general procedure (C): 4'-methoxychalcone **1t** (48 mg, 0.20 mmol) in triglyme (0.5 mL) was added slowly to the solution of KHMDS (80 mg, 0.4 mmol) and HCF_3_ (excess) in triglyme (0.5 mL) at –40 ^o^C. And the resulting mixture was stirred at the same temperature for 12 h. Isolated by column chromatography on silica gel (*n*-hexane/ethyl acetate = 95/5) to give **2t** (41 mg, 67% yield) as a colorless oil. ^1^H NMR (CDCl_3_, 300 MHz) δ 2.65 (s, 1H), 3.82 (s, 3H), 6.70 (d, *J* = 16.2 Hz, 1H), 6.85–6.94 (m, 3H), 7.29–7.38 (m, 3H), 7.42–7.44 (m, 2H), 7.56 (d, *J* = 8.7 Hz, 2H); ^19^F NMR (CDCl_3_, 282 MHz) δ −79.3 (s, 3F); MS (ESI, *m/z*) 307 [M−H]^−^

**(*E*)-2-(4-Chlorophenyl)-1,1,1-trifluoro-4-phenylbut-3-en-2-ol (2u)**

This compound has been previously prepared and characterized.^3^

(*E*)-2-(4-Chlorophenyl)-1,1,1-trifluoro-4-phenylbut-3-en-2-ol **2u** was prepared following general procedure (C): 4'-chlorochalcone **1u** (49 mg, 0.20 mmol) in triglyme (0.5 mL) was added slowly to the solution of KHMDS (80 mg, 0.4 mmol) and HCF_3_ (excess) in triglyme (0.5 mL) at –40 ^o^C. And the resulting mixture was stirred at the same temperature for 12 h. Isolated by column chromatography on silica gel (*n*-hexane/ethyl acetate = 98/2) to give **2u** (38 mg, 61% yield) as a colorless oil. ^1^H NMR (CDCl_3_, 300 MHz) δ 2.72 (s, 1H), 6.69 (d, *J* = 16.2 Hz, 1H), 6.86 (d, *J* = 16.2 Hz, 1H), 7.31–7.44 (m, 7H), 7.58 (d, *J* = 8.4 Hz, 2H); ^19^F NMR (CDCl_3_, 282 MHz) δ −79.3 (s, 3F); MS (ESI, *m/z*) 311 [M−H]^−^

**(*E*)-1,1,1-Trifluoro-2-(4-nitrophenyl)-4-phenylbut-3-en-2-ol (2v)**

(*E*)-1,1,1-Trifluoro-2-(4-nitrophenyl)-4-phenylbut-3-en-2-ol **2v** was prepared following general procedure (C): 4'-nitrochalcone **1v** (51 mg, 0.20 mmol) in triglyme (0.5 mL) was added slowly to the solution of KHMDS (80 mg, 0.4 mmol) and HCF_3_ (excess) in triglyme (0.5 mL) at –40 ^o^C. And the resulting mixture was stirred at the same temperature for 12 h. Isolated by column chromatography on silica gel (*n*-hexane/ethyl acetate = 95/5) to give **2v** (35 mg, 54% yield) as a yellow oil.

^1^H NMR (CDCl_3_, 300 MHz) δ 2.97 (s, 1H), 6.72 (d, *J* = 16.2 Hz, 1H), 6.87 (d, *J* = 16.2 Hz, 1H), 7.32–7.44 (m, 5H), 7.85 (d, *J* = 8.7 Hz, 2H), 8.23-8.27 (m, 2H); ^13^C NMR (CDCl_3_, 125.8 MHz) δ 77.1 (q, *J* = 29.7 Hz), 123.4, 124.5 (q, *J* = 286.4 Hz), 125.2, 126.9, 128.1, 128.8, 129.1, 134.8, 134.9, 144.1, 147.9; ^19^F NMR (CDCl_3_, 282 MHz) δ −79.1 (s, 3F); IR (neat) 3500, 1601, 1523, 1349, 1252, 1163, 1111, 1010, 973, 937, 854, 746, 713, 694 cm^−1^; MS (ESI, *m/z*) 322 [M−H]^−^, HRMS (ESI, *m/z*) [M−H]^−^ Calcd. for C_16_H_11_F_3_NO_3_ 322.0691: Found 322.0691.

**(*E*)-1,1,1-Trifluoro-2,4-bis(4-fluorophenyl)but-3-en-2-ol (2w)**

This compound has been previously prepared and characterized.^3^

*(E*)-1,1,1-Trifluoro-2,4-bis(4-fluorophenyl)but-3-en-2-ol **2w** was prepared following general procedure (C): 4,4'-difluorochalcone **1w** (45 mg, 0.20 mmol) in triglyme (0.5 mL) was added slowly to the solution of KHMDS (80 mg, 0.4 mmol) and HCF_3_ (excess) in triglyme (0.5 mL) at –40 ^o^C. And the resulting mixture was stirred at the same temperature for 12 h. Isolated by column chromatography on silica gel (*n*-hexane/ethyl acetate = 98/2) to give **2w** (46 mg, 73% yield) as a colorless oil. ^1^H NMR (CDCl_3_, 300 MHz) δ 2.71 (s, 1H), 6.61 (d, *J* = 16.2 Hz, 1H), 6.83 (d, *J* = 16.2 Hz, 1H), 7.01–7.12 (m, 4H), 7.40 (m, 2H), 7.62 (dd, *J* = 8.4, 5.4 Hz, 2H); ^19^F NMR (CDCl_3_, 282 MHz) δ −113.5–−113.4 (m, 1F), −113.0–−112.9 (m, 1F), −79.3 (m, 3F); MS (ESI, *m/z*) 313 [M−H]^−^

**5.4 General procedure (D):**

Trifluoromethylation of aryl aldehydes **1x**-**1z** and **1aa**-**1ee** by using triglyme as solvent in Scheme 2**.**

In glove box, a dried 10 mL vessel was successively charged with *^t^*BuOK(45 mg, 0.4 mmol) and dry triglyme (0.5 mL), and was sealed by a rubber septum and brought to bench. After being charged with fluoroform by cooling in liquid nitrogen under vacuum, the resulting mixture was warmed to −40 ^o^C. And a solution of carbonyl compounds **1i**-**n** and **1s-w** (0.20 mol) in triglyme (0.5 mL) was added slowly (over 5 min) by syringe. Then the reaction mixture was stirred at the same temperature for 12 h, quenched by addition of sat. NH_4_Cl aq., extracted with Et_2_O, dried over with Na_2_SO_4_ and then concentrated in vacuo. The residue was purified by column chromatography on silica gel (n-hexane/ethyl acetate) to give corresponding α-trifluoromethyl alcohols **2x**-**2z** and **2aa-2ee** in good yields.

**2,2,2-Trifluoro-1-(4-methoxyphenyl)ethan-1-ol (2x)**

This compound has been previously prepared and characterized.^3^

2,2,2-Trifluoro-1-(4-methoxyphenyl)ethan-1-ol **2x** was prepared following general procedure (D): 4-methoxybenzaldehyde **1x** (27 mg, 0.20 mmol) in triglyme (0.5 mL) was added slowly to the solution of *^t^*BuOK(45 mg, 0.4 mmol) and HCF_3_ (excess) in triglyme (0.5 mL) at –40 ^o^C. And the resulting mixture was stirred at the same temperature for 12 h. Isolated by column chromatography on silica gel (*n*-hexane/ethyl acetate = 85/15) to give **2x** (27 mg, 65% yield) as a colorless oil. ^1^H NMR (CDCl_3_, 300 MHz) δ 2.64 (brs, 1H), 3.82 (s, 3H), 4.96 (q, *J* = 6.5 Hz, 1H), 6.93 (d, *J* = 8.7 Hz, 2H), 7.39 (d, *J* = 8.7 Hz, 2H); ^19^F NMR (CDCl_3_, 282 MHz) δ −79.0 (d, *J* = 6.5 Hz, 3F); MS (ESI, *m/z*) 205 [M−H]^−^

**2,2,2-Trifluoro-1-phenylethan-1-ol (2y)**

This compound has been previously prepared and characterized.^1^

2,2,2-Trifluoro-1-phenylethan-1-ol **2y** was prepared following general procedure (D): benzaldehyde **1y** (21 mg, 0.20 mmol) in triglyme (0.5 mL) was added slowly to the solution of *^t^*BuOK ( 45 mg, 0.4 mmol) and HCF_3_ (excess) in triglyme (0.5 mL) at –40 ^o^C. And the resulting mixture was stirred at the same temperature for 12 h. Isolated by column chromatography on silica gel (*n*-hexane/ethyl acetate = 90/10) to give **2y** ( 20 mg, 57% yield) as a colorless oil. ^1^H NMR (CDCl_3_, 300 MHz) δ 2.68 (d, *J* = 4.2 Hz, 1H), 4.97–5.05 (m, 1H), 7.40–7.43 (m, 3H), 7.46–7.49 (m, 2H); ^19^F NMR (CDCl_3_, 282 MHz) δ −78.8 (d, *J* = 6.8 Hz, 3F); MS (ESI, *m/z*) 175 [M−H]^−^

**1-(4-(Dimethylamino)phenyl)-2,2,2-trifluoroethan-1-ol (2z)**

This compound has been previously prepared and characterized.^3^

1-(4-(Dimethylamino)phenyl)-2,2,2-trifluoroethan-1-ol **2z** was prepared following general procedure (D): 4-(dimethylamino)benzaldehyde **1z** (30 mg, 0.20 mmol) in triglyme (0.5 mL) was added slowly to the solution of *^t^*BuOK (45 mg, 0.4 mmol) and HCF_3_ (excess) in triglyme (0.5 mL) at –40 ^o^C. And the resulting mixture was stirred at the same temperature for 12 h. Isolated by column chromatography on silica gel (*n*-hexane/ethyl acetate = 85/15) to give **2z** (35 mg, 80% yield) as an orange solid.

^1^H NMR (CDCl_3_, 300 MHz) δ 2.39 (d, *J* = 4.2 Hz, 1H), 2.97 (s, 6H), 4.87–4.95 (m, 1H), 6.72 (d, *J* = 8.7 Hz, 2H), 7.32 (d, *J* = 8.4 Hz, 2H); ^19^F NMR (CDCl_3_, 282 MHz) δ −78.9 (d, *J* = 6.5 Hz, 3F); MS (ESI, *m/z*) 218 [M−H]^−^

**1-([1,1'-Biphenyl]-4-yl)-2,2,2-trifluoroethan-1-ol (2aa)**

This compound has been previously prepared and characterized.^3^

1-([1,1'-Biphenyl]-4-yl)-2,2,2-trifluoroethan-1-ol **2aa** was prepared following general procedure (D): [1,1'-biphenyl]-4-carbaldehyde **1aa** (37 mg, 0.20 mmol) in triglyme (0.5 mL) was added slowly to the solution of *^t^*BuOK(45 mg, 0.4 mmol) and HCF_3_ (excess) in triglyme (0.5 mL) at –40 ^o^C. And the resulting mixture was stirred at the same temperature for 12 h. Isolated by column chromatography on silica gel (*n*-hexane/ethyl acetate = 90/10) to give **2aa** (31 mg, 62% yield) as a white solid. ^1^H NMR (CDCl_3_, 300 MHz) δ 2.58 (d, *J* = 4.5 Hz, 1H), 5.05–5.13 (m, 1H), 7.35–7.40 (m, 1H), 7.44–7.49 (m, 2H), 7.54–7.66 (m, 6H); ^19^F NMR (CDCl_3_, 282 MHz) δ −78.8 (d, *J* = 6.8 Hz, 3F); MS (ESI, *m/z*) 251 [M−H]^−^

**1-(4-Bromophenyl)-2,2,2-trifluoroethan-1-ol (2bb)**

This compound has been previously prepared and characterized.^3^

1-(4-Bromophenyl)-2,2,2-trifluoroethan-1-ol **2bb** was prepared following general procedure (D) 4-bromobenzaldehyde **1bb** (37 mg, 0.20 mmol.dissolved in triglyme (0.5 mL) was added slowly to the solution of *^t^*BuOK(45 mg, 0.4 mmol) and HCF_3_ (excess) in triglyme (0.5 mL) at –40 ^o^C. And the resulting mixture was stirred at the same temperature for 12 h. Isolated by column chromatography on silica gel (*n*-hexane/ethyl acetate = 90/10) to give **2bb** (25 mg, 48% yield) as a colorless oil. ^1^H NMR (CDCl_3_, 300 MHz) δ 2.70 (brs, 1H), 5.00 (q, *J* = 6.5 Hz, 1H), 7.36 (d, *J* = 8.1 Hz, 2H), 7.55 (d, *J* = 8.4 Hz, 2H); ^19^F NMR (CDCl_3_, 282 MHz) δ −79.0 (d, *J* = 6.2 Hz, 3F); MS (ESI, *m/z*) 253 [M−H]^−^

**2,2,2-Trifluoro-1-(m-tolyl)ethan-1-ol (2cc)**

This compound has been previously prepared and characterized.^3^

2,2,2-Trifluoro-1-(m-tolyl)ethan-1-ol **2cc** was prepared following general procedure (D): 3-methylbenzaldehyde **1cc** (24 mg, 0.20 mmol) in triglyme (0.5 mL) was added slowly to the solution of , *^t^*BuOK(45 mg, 0.4 mmol) and HCF_3_ (excess) in triglyme (0.5 mL) at –40 ^o^C. And the resulting mixture was stirred at the same temperature for 12 h. Isolated by column chromatography on silica gel (*n*-hexane/ethyl acetate = 95/5) to give **2cc** (19 mg, 50% yield) as a colorless oil. ^1^H NMR (CDCl_3_, 300 MHz) δ 2.38 (s, 3H), 2.59 (brs, 1H), 4.98 (q, *J* = 6.7 Hz, 1H), 7.21–7.33 (m, 4H); ^19^F NMR (CDCl_3_, 282 MHz) δ −78.8 (d, *J* = 6.8 Hz, 3F); MS (ESI, *m/z*) 189 [M−H]^−^

**1-(Anthracen-9-yl)-2,2,2-trifluoroethan-1-ol (2dd)**

This compound has been previously prepared and characterized.^3^

1-(Anthracen-9-yl)-2,2,2-trifluoroethan-1-ol **2dd** was prepared following general procedure (D): anthracene-9-carbaldehyde **1dd** (41 mg, 0.20 mmol). dissolved in triglyme (0.5 mL) was added slowly to the solution of *^t^*BuOK(45 mg, 0.4 mmol) and HCF_3_ (excess) in triglyme (0.5 mL) at –40 ^o^C. And the resulting mixture was stirred at the same temperature for 12 h. Isolated by column chromatography on silica gel (*n*-hexane/ethyl acetate = 98/2) to give **2dd** (39 mg, 70% yield) as a yellow solid. ^1^H NMR (CDCl_3_, 300 MHz) δ 3.00 (d, *J* = 4.2 Hz, 1H), 6.67 (qd, *J* = 7.9, 4.2 Hz, 1H), 7.47–7.52 (m, 2H), 7.57 (t, *J =* 7.5 Hz, 2H), 8.04 (d, *J* = 8.1 Hz, 2H), 8.15 (brs, 1H), 8.55 (s, 1H), 8.95 (brs, 1H); ^19^F NMR (CDCl_3_, 282 MHz) δ −74.5 (d, *J* = 7.9 Hz, 3F); MS (ESI, *m/z*) 275 [M−H]^−^

**2,2,2-Trifluoro-1-(furan-2-yl)ethan-1-ol (2ee)**

This compound has been previously prepared and characterized.^3^ This compound (**2ee**) is volatile and difficult to isolate. ^19^F NMR yield is 48% with PhCF_3_ as an internal standard.

2,2,2-Trifluoro-1-(furan-2-yl)ethan-1-ol **2ee** was prepared following general procedure (D) furfural **1ee** (19 mg, 0.20 mmol) in triglyme (0.5 mL) was added slowly to the solution of *^t^*BuOK(45 mg, 0.4 mmol) and HCF_3_ (excess) in triglyme (0.5 mL) at –40 ^o^C. And the resulting mixture was stirred at the same temperature for 12 h. Isolated by column chromatography on silica gel *n*-hexane/ethyl acetate = 80/20) to give **2ee** (15 mg, 44% yield) as a colorless oil. ^1^H NMR (CDCl_3_, 300 MHz) δ 2.79 (brs, 1H), 5.06 (q, *J* = 6.5 Hz, 1H), 6.42–6.44 (m, 1H), 6.54 (d, *J* = 3.3 Hz, 1H), 7.47–7.48 (m, 1H); ^19^F NMR (CDCl_3_, 282 MHz) δ −78.5 (d, *J* = 6.5 Hz, 3F); MS (ESI, *m/z*) 165 [M−H]^−^

**5.5 General procedure (E):**

Trifluoromethylation of enolizable ketones **1ff**-**1kk** in the presence of 18-crown-6 in Scheme 3

In glove box, a dried 10 mL vessel was successively charged with *^t^*BuOK (67 mg, 0.6 mmol), 18-crown-6 (159 mg, 0.6 mmol) and THF (2.0 mL), and was sealed by a rubber septum and brought to bench. Subsequently, the solution was cooled in liquid nitrogen followed by adding carbonyl compounds (enolizable ketones **1ff-1kk**) under argon atmosphere. Then the resulting mixture was charged with HCF_3_ by cooling at the same temperature under vacuum. Then the solution was allowed to warm to room temperature. After being stirred for 6-12 h monitoring by TLC upon the completion of the reaction, the resulting mixture was quenched with sat. NH_4_Cl aq. extracted with Et_2_O, dried over with Na_2_SO_4_ and then concentrated in vacuo. The residue was purified by column chromatography on silica gel (n-hexane/ethyl acetate) to give corresponding α-trifluoromethyl alcohols **2ff-2kk** in good yields.

**1,1,1-Trifluoro-2-(4-methoxyphenyl)propan-2-ol (2ff)**

This compound has been previously prepared and characterized.^7^

1,1,1-Trifluoro-2-(4-methoxyphenyl)propan-2-ol **2ff** was prepared following general procedure (E): the reaction of 1-(4-methoxyphenyl)ethan-1-one **1ff** (30 mg, 0.20 mmol). with HCF_3_ (excess) was carried out in the presence of *^t^*BuOK (67 mg, 0.6 mmol) and 18-crown-6 (159 mg, 0.6 mmol) in THF (2.0 mL) at room temperature for 12 h. Isolated by column chromatography on silica gel (*n*-hexane/ethyl acetate = 90/10) to give **2ff** (33 mg, 76% yield) as a colorless oil. ^1^H NMR (CDCl_3_, 300 MHz) δ 1.77 (s, 3H), 2.40 (s, 1H), 3.82 (s, 3H), 6.92 (d, *J* = 9.0 Hz, 2H), 7.50 (d, *J* = 8.7 Hz, 2H); ^19^F NMR (CDCl_3_, 282 MHz) δ −81.7 (s, 3F); MS (ESI, *m/z*) 219 [M−H]^−^

**1,1,1-Trifluoro-2-(3-methoxyphenyl)propan-2-ol (2gg)**

This compound has been previously prepared and characterized.^7^

1,1,1-Trifluoro-2-(3-methoxyphenyl)propan-2-ol **2gg** was prepared following general procedure (E): the reaction of 3'-methoxyacetophenone **1gg** (30 mg, 0.20 mmol) with HCF_3_ (excess) was carried out in the presence of *^t^*BuOK (67 mg, 0.6 mmol) and 18-crown-6 (159 mg, 0.6 mmol) in THF (2.0 mL) at room temperature for 12 h. Isolated by column chromatography on silica gel (*n*-hexane/ethyl acetate = 90/10) to give **2gg** (15 mg, 34% yield) as a colorless oil. ^1^H NMR (CDCl_3_, 300 MHz) δ 1.77 (s, 3H), 2.43 (s, 1H), 3.83 (s, 3H), 6.89–6.93 (m, 1H), 7.13–7.15 (m, 2H), 7.29–7.35 (m, 1H); ^19^F NMR (CDCl_3_, 282 MHz) δ −81.3 (s, 3F); MS (ESI, *m/z*) 219 [M−H]^−^

**1,1,1-Trifluoro-2-(2-methoxyphenyl)propan-2-ol (2hh)**

This compound has been previously prepared and characterized.^9^

1,1,1-Trifluoro-2-(2-methoxyphenyl)propan-2-ol **2hh** was prepared following general procedure (E): the reaction of 2'-methoxyacetophenone **1hh** (30 mg, 0.20 mmol) with HCF_3_ (excess) was carried out in the presence of *^t^*BuOK (67 mg, 0.6 mmol) and 18-crown-6 (159 mg, 0.6 mmol) in THF (2.0 mL) at room temperature for 12 h. Isolated by column chromatography on silica gel (*n*-hexane/ethyl acetate = 95/5) to give **2hh** (23 mg, 52% yield) as a colorless oil. ^1^H NMR (CDCl_3_, 300 MHz) δ 1.77 (s, 3H), 3.94 (s, 3H), 6.13 (s, 1H), 6.99–7.05 (m, 2H), 7.31–7.39 (m, 2H); ^19^F NMR (CDCl_3_, 282 MHz) δ −81.8 (s, 3F); MS (ESI, *m/z*) 243 [M+Na]^+^

**1,1,1-Trifluoro-2-(p-tolyl)propan-2-ol (2ii)**

This compound has been previously prepared and characterized.^8^

1,1,1-Trifluoro-2-(p-tolyl)propan-2-ol **2ii** was prepared following general procedure (E): the reaction of 4'-methylacetophenone **1ii** (27 mg, 0.20 mmol). with HCF_3_ (excess) was carried out in the presence of *^t^*BuOK (67 mg, 0.6 mmol) and 18-crown-6 (159 mg, 0.6 mmol) in THF (2.0 mL) at room temperature for 12 h. Isolated by column chromatography on silica gel (*n*-hexane/ethyl acetate = 95/5) to give **2ii** (20 mg, 49% yield) as a colorless oil. ^1^H NMR (CDCl_3_, 300 MHz) δ 1.77 (s, 3H), 2.36 (s, 3H), 2.39 (s, 1H), 7.21 (d, *J* = 8.1 Hz, 2H), 7.46 (d, *J* = 7.8 Hz, 2H); ^19^F NMR (CDCl_3_, 282 MHz) δ −81.5 (s, 3F); MS (ESI, *m/z*) 203 [M−H]^−^

**1,1,1-Trifluoro-2-(naphthalen-2-yl)propan-2-ol (2jj)**

This compound has been previously prepared and characterized.^8^

1,1,1-Trifluoro-2-(naphthalen-2-yl)propan-2-ol **2jj** was prepared following general procedure (E): the reaction of 4'-methylacetophenone **1jj** (34 mg, 0.20 mmol) with HCF_3_ (excess) was carried out in the presence of *^t^*BuOK (67 mg, 0.6 mmol) and 18-crown-6 (158.6 mg, 0.6 mmol) in THF (2.0 mL) at room temperature for 12 h. Isolated by column chromatography on silica gel (*n*-hexane/ethyl acetate = 90/10) to give **2jj** (17 mg, 35% yield) as a white solid. ^1^H NMR (CDCl_3_, 300 MHz) δ 1.88 (s, 3H), 2.63 (br s, 1H), 7.49–7.54 (m, 2H), 7.67 (d, *J* = 8.7 Hz, 1H), 7.83–7.89 (m, 3H), 8.07 (s, 1H); ^19^F NMR (CDCl_3_, 282 MHz) δ −81.1 (s, 3F); MS (ESI, *m/z*) 273 [M+CH_3_OH_2_]^+^

**1,1,1-Trifluoro-2-(4-methoxyphenyl)butan-2-ol (2kk)**

This compound has been previously prepared and characterized.^10^

1,1,1-Trifluoro-2-(4-methoxyphenyl)butan-2-ol **2kk** was prepared following general procedure (E): the reaction of 1-(4-methoxyphenyl)propan-1-one **1kk** (33 mg, 0.20 mmol) with HCF_3_ (excess) was carried out in the presence of *^t^*BuOK (67 mg, 0.6 mmol) and 18-crown-6 (159 mg, 0.6 mmol) in THF (2.0 mL) at room temperature for 12 h. Isolated by column chromatography on silica (*n*-hexane/ethyl acetate = 95/5) to give **2kk** (27 mg, 58% yield) as a colorless oil. ^1^H NMR (CDCl_3_, 300 MHz) δ 0.80 (t, *J* = 7.4 Hz, 3H), 1.96–2.28 (m, 2H), 2.31 (s, 1H), 3.82 (s, 3H), 6.92 (d, *J* = 9.0 Hz, 2H), 7.44 (d, *J* = 8.7 Hz, 2H); ^19^F NMR (CDCl_3_, 282 MHz) δ −80.8 (s, 3F); MS (ESI, *m/z*) 233 [M−H]^−^

**Reference:**

1. S. Okusu, K. Hirano, E. Tokunaga and N. Shibata, *ChemistryOpen*, 2015, **4**, 581-585.
2. G. K. S. Prakash, P. V. Jog, P. T. D. Batamack and G. A. Olah, *Science*, 2012, **338**, 1324-1327.
3. K. Aikawa, W. Toya, Y. Nakamura and K. Mikami, *Org. Lett.*, 2015, **17**, 4996-4999.
4. L. Jablonski, J. Joubert, T. Billard and B. R. Langlois, *Synlett*, 2003, 230-232.
5. M. J. O’Connor, K. N. Boblak, M. J. Topinka, P. J. Kindelin, J. M. Briski, C. Zheng and D. A. Klumpp, *J. Am. Chem. Soc.*, 2010, **132**, 3266-3267.
6. G. K. S. Prakash, Z. Zhang, F. Wang, S. Munoz and G. A. Olah, *J. Org. Chem.*, 2013, **78**, 3300-3305.
7. S. Mizuta, N. Shibata, S. Akiti, H. Fujimoto, S. Nakamura and T. Toru, *Org. Lett.*, 2007, **9**, 3707-3710.
8. X. Hu, J. Wang, W. Li, L. Lin, X. Liu and X. Feng, *Tetrahedron Lett.*, 2009, **50**, 4378-4380.
9. M. Calvillo-Barahona, C. Cordovilla, M. N. Genov, J. M. Martinez-Ilarduya and P. Espinet, *Dalton Trans.*, 2013, **42**, 14576-14582.
10. K. Yearick and C. Wolf, *Org. Lett.*, 2008, **10**, 3915-3918.

# **6. Copies of ^1^H, ^13^Cand ^19^F NMR spectra of new compounds 2g, 2k, 2m, 2n, 2v**

**1,1-Bis(4-bromophenyl)-2,2,2-trifluoroethanol (2g)**

**2,2,2-Trifluoro-1-(furan-2-yl)-1-phenylethan-1-ol (2k)**

**2,2,2-Trifluoro-1-phenyl-1-(pyridin-3-yl)ethan-1-ol (2m)**


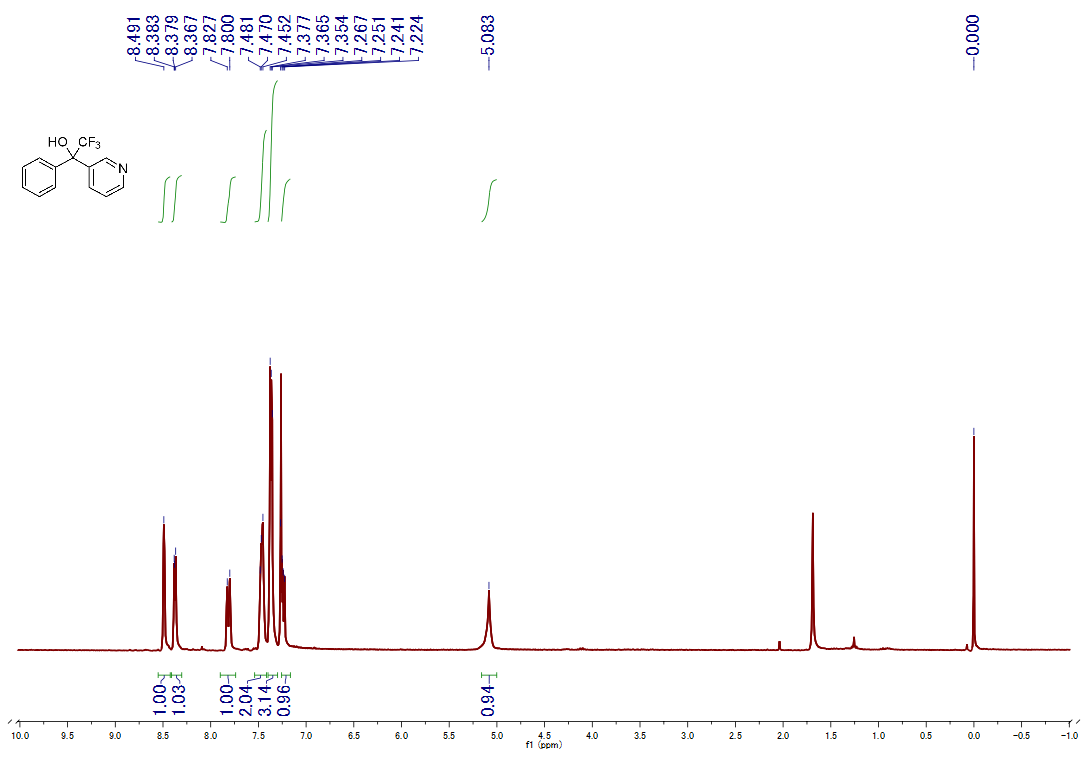

**2,2,2-Trifluoro-1-phenyl-1-(pyridin-4-yl)ethan-1-ol (2n)**

**(*E*)-1,1,1-Trifluoro-2-(4-nitrophenyl)-4-phenylbut-3-en-2-ol (2v)**

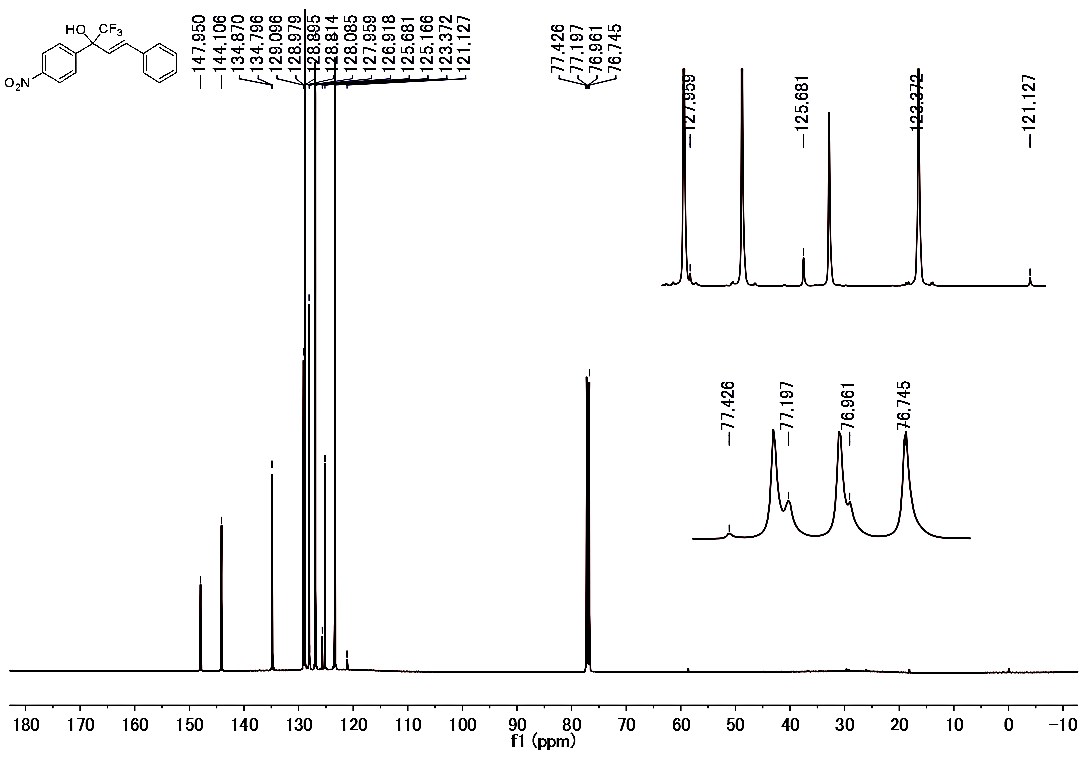

**7. Copies of ^1^H and ^19^F NMR spectra for known compounds 2a-2f, 2h-2j, 2l, 2o-2u, 2w-2z, 2aa-2kk.**

**2,2,2-Trifluoro-1,1-diphenylethanol (2a)**

**2,2,2-Trifluoro-1-(4-methoxyphenyl)-1-phenylethanol (2b)**

**2,2,2-Trifluoro-1-phenyl-1-p-tolylethanol (2c)**

**1,1-Bis(4-chlorophenyl)-2,2,2-trifluoroethanol (2d)**

**1-(3-Chlorophenyl)-2,2,2-trifluoro-1-phenylethanol (2e)**

**2,2,2-Trifluoro-1-(4-fluorophenyl)-1-phenylethanol (2f)**

**2,2,2-Trifluoro-1-(4-(trifluoromethyl)phenyl)-1-phenylethanol (2h)**

**2,2,2-Trifluoro-1-(4-nitrophenyl)-1-phenylethan-1-ol (2i)**

**2,2,2-Trifluoro-1-phenyl-1-(thiophen-2-yl)ethan-1-ol (2j)**

**2,2,2-Trifluoro-1-phenyl-1-(pyridin-2-yl)ethan-1-ol (2l)**

**9-(Trifluoromethyl)-9H-fluoren-9-ol (2o)**

**9-(Trifluoromethyl)-9H-xanthen-9-ol (2p)**

**2-(Trifluoromethyl)-2-adamantanol (2q)**

**1,1,1-Trifluoro-3,3-dimethyl-2-phenylbutan-2-ol (2r)**

**(*E*)-1,1,1-Trifluoro-2,4-diphenylbut-3-en-2-ol (2s)**

**(*E*)-1,1,1-Trifluoro-2-(4-methoxyphenyl)-4-phenylbut-3-en-2-ol (2t)**

**(*E*)-2-(4-Chlorophenyl)-1,1,1-trifluoro-4-phenylbut-3-en-2-ol (2u)**

**(*E*)-1,1,1-Trifluoro-2,4-bis(4-fluorophenyl)but-3-en-2-ol (2w)**

**2,2,2-Trifluoro-1-(4-methoxyphenyl)ethan-1-ol (2x)**

**2,2,2-Trifluoro-1-phenylethan-1-ol (2y)**

**1-(4-(Dimethylamino)phenyl)-2,2,2-trifluoroethan-1-ol (2z)**

**1-([1,1'-Biphenyl]-4-yl)-2,2,2-trifluoroethan-1-ol (2aa)**

**1-(4-**B**romophenyl)-2,2,2-trifluoroethan-1-ol (2bb)**

**2,2,2-Trifluoro-1-(m-tolyl)ethan-1-ol (2cc)**

**1-(Anthracen-9-yl)-2,2,2-trifluoroethan-1-ol (2dd)**

**2,2,2-Trifluoro-1-(furan-2-yl)ethan-1-ol (2ee)**

**1,1,1-Trifluoro-2-(4-methoxyphenyl)propan-2-ol (2ff)**

**1,1,1-Trifluoro-2-(3-methoxyphenyl)propan-2-ol (2gg)**

**1,1,1-Trifluoro-2-(2-methoxyphenyl)propan-2-ol (2hh)**

**1,1,1-Trifluoro-2-(p-tolyl)propan-2-ol (2ii)**

**1,1,1-Trifluoro-2-(naphthalen-2-yl)propan-2-ol (2jj)**

**1,1,1-Trifluoro-2-(4-methoxyphenyl)butan-2-ol (2kk)**

# **8. Computational methods**

**The optimized structures of [K(triglyme)_2_][CF_3_], [K(tetraglyme)_2_][CF_3_], [K(18-crown-6)/(THF)_2_] [CF_3_], [K][CF_3_] in (Figure 1).**

Gaussian09 program ^1^ was used for DFT calculations. The geometries of complexes were optimized at the B3LYP/6-311G** level. ^2,3^ The intermolecular interaction energies for complexes (*E*_int_) were calculated at the B3LYP/6-311G** level by the supermolecule method. The basis set superposition error (BSSE)^4^ was corrected for all the interaction energy calculations using the counterpoise method.^5^ The stabilization energy for forming a complex from isolated species (*E*_form_) was calculated as the sum of the *E*_int_ and the deformation energy (*E*_def_), which is the sum of the increases in energy of molecules due to the deformation during formation of the complex. Here, the *E*_def_ was calculated at the B3LYP/6-311G** level.

The optimized structures of the complexes [K(triglyme)_2_][CF_3_], [K(tetraglyme)_2_][CF_3_], [K(18-crown-6)][CF_3_], [K(18-crown-6)/(THF)][CF_3_] and KCF_3_ was shown as follow (**Table S5-S13**).

**Table S5:** Minima for [K(G3)_2_][CF_3_] and their stabilization energy (*E_from_*) in kcal/mol.


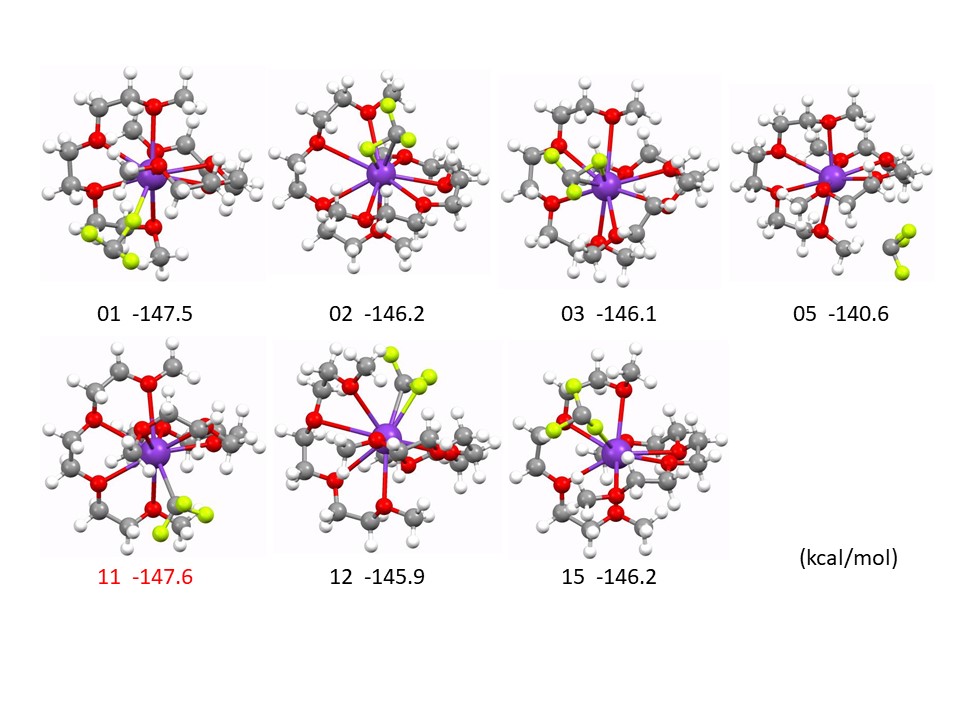


**Table S6:** Minima for [K(G4)_2_][CF_3_] and their stabilization energy (*E_from_*) in kcal/mol.


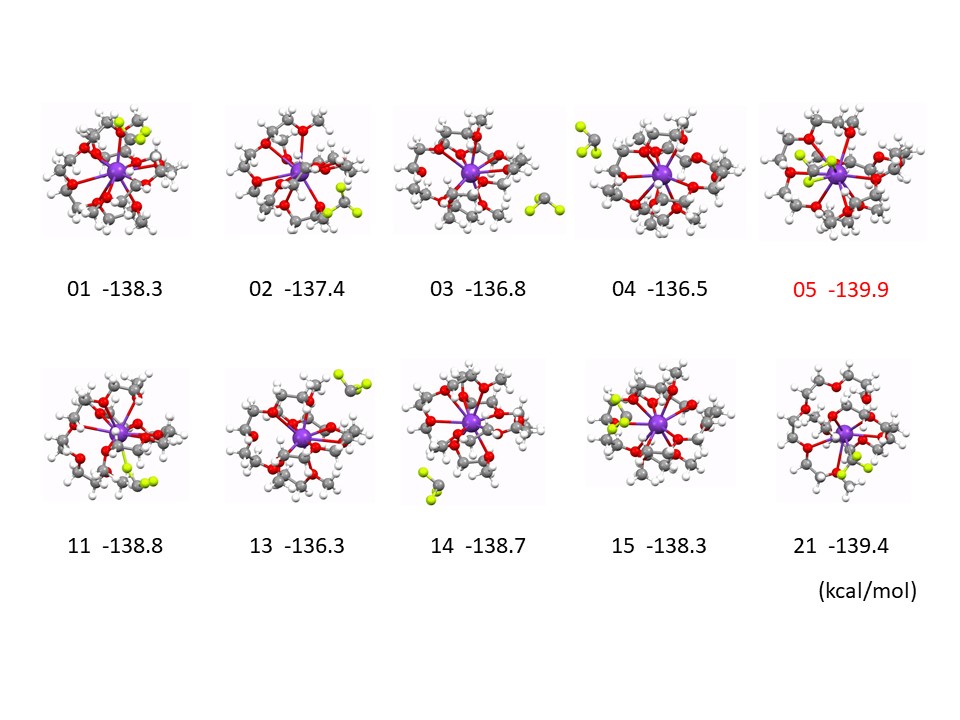


**Table S7**: Optimized structure of [K(triglyme)_2_][CF_3_] (**b** in Figure 2)

| [K(triglyme)_2_][CF_3_] | | | | |
| --- | --- | --- | --- | --- |
| atom | distance (Å) |  | atom | angle (°) |
| C-F1 | 1.415 |  | F1-C-F2 | 100.66 |
| C-F2 | 1.432 |  | F2-C-F3 | 99.68 |
| C-F3 | 1.441 |  | F3-C-F1 | 100.33 |
| K-F1 | 3.893 |  | K-C-F1 | 119.90 |
| K-F2 | 3.984 |  | K-C-F2 | 124.76 |
| K-F3 | 3.684 |  | K-C-F3 | 107.29 |
| K-C | 2.990 |  |  |  |

**Table S8**: Optimized structure of [K(triglyme)_2_][CF_3_] disordered isomer (**c** in Figure 2)


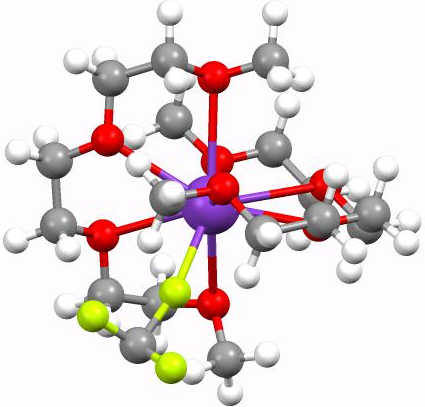


| [K(triglyme)_2_][CF_3_] disordered isomer | | | | |
| --- | --- | --- | --- | --- |
| atom | distance (Å) |  | atom | angle (°) |
| C-F1 | 1.422 |  | F1-C-F2 | 97.90 |
| C-F2 | 1.531 |  | F2-C-F3 | 98.66 |
| C-F3 | 1.391 |  | F3-C-F1 | 101.10 |
| K-F1 | 4.400 |  | K-C-F1 | 97.49 |
| K-F2 | 2.542 |  | K-C-F2 | 14.13 |
| K-F3 | 4.694 |  | K-C-F3 | 112.47 |
| K-C | 3.983 |  |  |  |

**Table S9**: Optimized structure of [K(tetraglyme)_2_][CF_3_] (**d** in Figure 2)

| [K(tetraglyme)_2_][CF_3_], | | | | |
| --- | --- | --- | --- | --- |
| atom | distance (Å) |  | atom | angle (°) |
| C-F1 | 1.425 |  | F1-C-F2 | 100.54 |
| C-F2 | 1.428 |  | F2-C-F3 | 99.70 |
| C-F3 | 1.440 |  | F3-C-F1 | 100.01 |
| K-F1 | 3.942 |  | K-C-F1 | 120.67 |
| K-F2 | 3.637 |  | K-C-F2 | 103.86 |
| K-F3 | 4.057 |  | K-C-F3 | 127.17 |
| K-C | 3.020 |  |  |  |

**Table S10**: Optimized structure of major disordered isomer of [K(tetraglyme)_2_][CF_3_] (**e** in Figure 2)

**
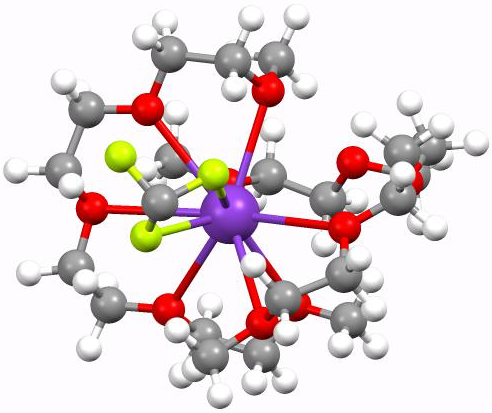
**

| [K(tetraglyme)_2_][CF_3_], | | | | |
| --- | --- | --- | --- | --- |
| atom | distance (Å) |  | atom | angle (°) |
| C-F1 | 1.395 |  | F1-C-F2 | 99.36 |
| C-F2 | 1.495 |  | F2-C-F3 | 97.10 |
| C-F3 | 1.469 |  | F3-C-F1 | 99.94 |
| K-F1 | 4.085 |  | K-C-F1 | 104.88 |
| K-F2 | 2.718 |  | K-C-F2 | 47.38 |
| K-F3 | 2.784 |  | K-C-F3 | 49.72 |
| K-C | 3.498 |  |  |  |

**Table S11**: Optimized structure of [K(18-crown-6)][CF_3_] in THF solution (**f** in Figure 2)

| [K(18-crown-6)][CF_3_] | | | | |
| --- | --- | --- | --- | --- |
| atom | distance (Å) |  | atom | angle (°) |
| C-F1 | 1.408 |  | F1-C-F2 | 99.60 |
| C-F2 | 1.460 |  | F2-C-F3 | 98.35 |
| C-F3 | 1.457 |  | F3-C-F1 | 99.67 |
| K-F1 | 3.863 |  | K-C-F1 | 96.73 |
| K-F2 | 2.740 |  | K-C-F2 | 50.13 |
| K-F3 | 2.718 |  | K-C-F3 | 49.19 |
| K-C | 3.436 |  |  |  |

**Table S12**: Optimized structure of [K(18-crown-6)/THF][CF_3_] in THF solution (**g** in Figure 2)

| [K(18-crown-6)/THF][CF_3_] | | | | |
| --- | --- | --- | --- | --- |
| atom | distance (Å) |  | atom | angle (°) |
| C-F1 | 1.422 |  | F1-C-F2 | 99.39 |
| C-F2 | 1.453 |  | F2-C-F3 | 98.53 |
| C-F3 | 1.445 |  | F3-C-F1 | 99.59 |
| K-F1 | 3.865 |  | K-C-F1 | 92.82 |
| K-F2 | 2.770 |  | K-C-F2 | 47.94 |
| K-F3 | 2.886 |  | K-C-F3 | 52.66 |
| K-C | 3.524 |  |  |  |

**Table S13**: Optimized structures (distance in Å and angle in °) of KCF_3_  (**h** in Figure 2)

| KCF_3_ | | | | |
| --- | --- | --- | --- | --- |
| atom | distance (Å) |  | atom | angle (°) |
| C-F1 | 1.343 |  | F1-C-F2 | 102.16 |
| C-F2 | 1.489 |  | F2-C-F3 | 97.01 |
| C-F3 | 1.489 |  | F3-C-F1 | 102.16 |
| K-F1 | 3.920 |  | K-C-F1 | 168.92 |
| K-F2 | 2.533 |  | K-C-F2 | 70.89 |
| K-F3 | 2.533 |  | K-C-F3 | 70.89 |
| K-C | 2.593 |  |  |  |

**Reference:**

1. Gaussian 09, Revision C.01, M. J. Frisch, G. W. Trucks, H. B. Schlegel, G. E. Scuseria, M. A. Robb, J. R. Cheeseman, G. Scalmani, V. Barone, B. Mennucci, G. A. Petersson, H. Nakatsuji, M. Caricato, X. Li, H. P. Hratchian, A. F. Izmaylov, J. Bloino, G. Zheng, J. L. Sonnenberg, M. Hada, M. Ehara, K. Toyota, R. Fukuda, J. Hasegawa, M. Ishida, T. Nakajima, Y. Honda, O. Kitao, H. Nakai, T. Vreven, J. A. Montgomery, Jr., J. E. Peralta, F. Ogliaro, M. Bearpark, J. J. Heyd, E. Brothers, K. N. Kudin, V. N. Staroverov, R. Kobayashi, J. Normand, K. Raghavachari, A. Rendell, J. C. Burant, S. S. Iyengar, J. Tomasi, M. Cossi, N. Rega, J. M. Millam, M. Klene, J. E. Knox, J. B. Cross, V. Bakken, C. Adamo, J. Jaramillo, R. Gomperts, R. E. Stratmann, O. Yazyev, A. J. Austin, R. Cammi, C. Pomelli, J. W. Ochterski, R. L. Martin, K. Morokuma, V. G. Zakrzewski, G. A. Voth, P. Salvador, J. J. Dannenberg, S. Dapprich, A. D. Daniels, Ö. Farkas, J. B. Foresman, J. V. Ortiz, J. Cioslowski, and D. J. Fox, Gaussian, Inc., Wallingford CT, 2009.
2. C. Lee, W. Yang, and R. G. Parr, Phys. Rev. B 1988, ***37***, 785-789.
3. A. D. Becke, J. Chem. Phys. 1993, ***98***, 5648-5652.
4. B. J. Ransil, *J. Chem. Phys*., 1961, **34***,* 2109-2118.
5. S. F. Boys, F. Bernardi, *Mol. Phys*., 1970*,* **19***,* 553-566.

1. Department of Nanopharmaceutical Sciences, Nagoya Institute of Technology, Gokiso, Showa-ku, Nagoya 466-5888, Japan [↑](#footnote-ref-1)
2. Research Center for Computational Design of Advanced Functional Materials, AIST, Tsukuba, Ibaraki 305-8568, Japan [↑](#footnote-ref-2)
3. Institute of Advanced Fluorine-Containing Materials, Zhejiang Normal University, 688 Yingbin Avenue, 321004 Jinhua, China [↑](#footnote-ref-3)
